# Paediatric chronic pain prevalence in low- and middle-income countries: A systematic review and meta-analysis

**DOI:** 10.1016/j.eclinm.2022.101296

**Published:** 2022-02-12

**Authors:** Zi Wei Liao, Chi Le, J. Matthew Kynes, Jonathan A. Niconchuk, Emilia Pinto, Heather E. Laferriere, Camila B. Walters

**Affiliations:** a 224 Eskind Biomedical Library and Learning Center, Vanderbilt University School of Medicine, Nashville, TN 37240-7939, USA; b Vanderbilt University Medical Center, 2200 Children's Way, Nashville, TN 37232, USA; c Maputo Central Hospital, 1653 Avenida Eduardo Mondlane, Maputo, Mozambique; d 224 Eskind Biomedical Library and Learning Center, Vanderbilt University, Nashville, TN 37240-7939, USA

**Keywords:** Chronic pain, Children, Adolescents, Paediatric pain, Pediatric pain, Low- and middleincome countries

## Abstract

**Background:**

Chronic pain is a leading cause of morbidity in children and adolescents globally, with a significant impact on quality of life. This is the first systematic review and meta-analysis on paediatric chronic pain in low- and middle-income countries (LMICs).

**Methods:**

Following the Preferred Reporting Items for Systematic Reviews and Meta-Analyses (PRISMA) guidelines, we searched MEDLINE (via PubMed), Embase, CINAHL, PsycINFO, Web of Science, Cochrane Database of Systematic Reviews, and the WHO Global Index Medicus for all studies published prior to January 7, 2022. Articles published in all languages that included populations age 19 years and under living in LMICs were considered. Chronic pain was defined as persistent or recurrent pain that is present for ≥3 months, per the International Classification of Diseases (ICD-11) definition. Summary data were extracted from published reports and evaluated with mixed-effects regression analysis. PROSPERO Record ID: CRD42021227967.

**Findings:**

Of the 2875 studies identified, 70 articles were reviewed, with 27 studies representing 20 LMICs eligible for analysis. The average prevalence for each pain type reported with 95% confidence interval is as follows: general/multi-site/any 20% (16–25), musculoskeletal (MSK) pain 9% (7–13), abdominal pain 7% (5–10), headache 4% (2–10), and fibromyalgia per American College of Rheumatology or Yunus and Masi criteria 3% (1–10). Overall, a pooled mean of 8% chronic pain was estimated across all studies. A significantly high level of heterogeneity was found across all studies (*I^2^* >90%). Chronic headache (OR=1·65, 95% CI 1·39–1·96), abdominal pain (OR=1·36, 95% CI 1·22–1·51), and generalized/multi-site pain (OR=1·54, 95% CI 1·31–1·81) were significantly more prevalent in females than males.

**Interpretation:**

The characterization of paediatric chronic pain in low- and middle-income countries suffers from a paucity of data and significant heterogeneity in the assessment methods. Understanding the global burden of chronic pain in this group should be prioritized.

**Funding:**

None.

**Research in context**

**Evidence before this study**

We searched for articles in all languages on the prevalence of chronic pain in children and adolescents age ≤19 years living in low- and middle-income countries (LMICs) on Pub MEDLINE (via PubMed), Embase (OvidSP), CINAHL (EBSCOhost), PsycINFO (ProQuest), Web of Science (Clarivate), Cochrane Database of Systematic Reviews (Wiley), and WHO Global Index Medicus from journal inception to January 7, 2022 and found no study prior to this study has evaluated the prevalence of chronic paediatric pain in LMICs as a systematic review and meta-analysis . Globally, only one systematic review that included mostly high-income countries (HICs) has been published (2011), with no meta-analysis on the topic. In HICs, the prevalence of paediatric chronic pain, based on individual studies, was estimated at 20% to 35%.

**Added value of this study**

To our knowledge, this is the first systematic review and meta-analysis on paediatric chronic pain in LMICs. Our results show there is a paucity of data on paediatric chronic pain and the available data suffers from significant heterogeneity in the assessment of pain, even with the use of the standardized chronic pain definition per the International Association for the Study of Pain. This study offers a summary of currently available evidence and will encourage researchers to use more standardized methods to obtain these data in the future, as having data on the status of paediatric chronic pain in low-resource settings is the first crucial step to relieve suffering in this population.

**Implications of all the available evidence**

The heterogeneity in chronic pain research in LMICs can confuse public health officials and undermine the importance of treating chronic pain in children and adolescents. Children with chronic pain can grow up to be adults with chronic pain, leading to decreased functionality and productivity, as well as poor quality of life. Future studies should focus on the development of culturally appropriate assessment tools to monitor and characterize paediatric chronic pain as the first-step to treating chronic pain in children and adolescents living in low-resource settings.

## Introduction

Chronic pain is a complex health condition with profound impact on quality of life, rates of disability, and economic well-being of families and communities.^1^ It has been estimated that 20% of adults globally suffer from chronic pain.^2^ In low- and middle-income countries (LMICs), the prevalence is higher at 33%.^2^^,^^3^ Prior estimates of the prevalence of chronic pain in adolescents and children, based largely on studies in high-income countries (HICs), has been posited at 20% to 35%.^4^, ^5^, ^6^ The latest systematic review on the epidemiology of paediatric chronic pain, however, was published a decade ago.^7^^,^^8^ There has been no published meta-analysis on global paediatric chronic pain prevalence, and no systematic review and meta-analysis has characterized the prevalence of chronic pain in adolescents and children living in LMICs.

Paediatric pain has significant long-term negative sequelae as it can hinder the educational, social, and physical development of a youth, along with mental health ramifications. The lack of data on paediatric chronic pain may reflect a lack of recognition of this phenomenon. Chronic pain is highly associated with disability^9^^,^^10^ In older children and adolescents, low back pain, neck pain, and migraines are the leading causes of years lived with disability.^10^ Children with undertreated chronic pain become adults with chronic pain.^11^, ^12^, ^13^ Unlike in HICs, where paediatric chronic pain is managed by a multi-disciplinary team with pain management training, in LMICs pain treatment capacity is limited and access to pain medications, invasive procedures, psychological therapy, physical therapy, and complimentary therapy (such as yoga and acupuncture) is often unavailable or inadequate. This disparity exists even though access to adequate pain treatment is “a fundamental human right,” and countries must take reasonable steps to fulfill their obligation towards those suffering with chronic pain.^14^

One of the first steps in providing adequate pain treatment is to describe the prevalence of paediatric chronic pain in LMICs. To that end, this systematic review and meta-analysis is intended to characterize the current literature on the prevalence of paediatric chronic pain in LMICs.

## Methods

The full protocol of this systematic review and meta-analysis was registered on the international prospective register of systematic reviews (PROSPERO, Record ID CRD42021227967) with two amendments.^15^ Per Preferred Reporting Items for Systematic Reviews and Meta-Analyses (PRISMA) guidelines, we searched MEDLINE (via PubMed), Embase (OvidSP), CINAHL (EBSCOhost), PsycINFO (ProQuest), Web of Science (Clarivate), Cochrane Database of Systematic Reviews (Wiley), and WHO Global Index Medicus in January 2022 using search terms in four conceptual blocks: (i) pain terms (“recurrent” or “recurring” or “persistent”, “pain”, “painful”, “ache”, “backache”, “headache”); (ii) paediatric terms (“neonates”, “paediatric/paediatric”, “child/children”, “adolescent”, “boy”, “girl”); (iii) geographical terms (“low income countries or middle income countries”, “LMIC”, “Africa”, “Asia”, “Central America”, “Latin America”, “South America”, names of individual countries); (iv) epidemiological terms (“prevalence”, “incidence”, epidemiology”). Search terms were formatted as needed for each database. The first amendment to the protocol was that African Journal Online and Bioline were hand-searched for completeness and did not yield any eligible studies. The reference lists of included studies were searched by hand to identify any additional articles. Limitations were added to limit to human studies and to exclude review articles, case studies, and conference abstracts. Individual country names were cross-referenced with The World Bank list of countries by income level. LMICs are defined as countries with a gross national income (GNI) per capita less than $12,535 in 2019 calculated using the *World Bank Atlas* method.^16^ A detailed search strategy is provided in Appendix 1.

### Inclusion and exclusion criteria

All articles published on populations age 19 and under in LMICs up to January 7, 2022, were included. All languages were included; articles not in English were translated via Google Translate. If articles were not readily available, full-text articles were requested from authors by direct contact. Two independent reviewers (ZL, CL, or CW) reviewed each article and title for relevance. Cases of disagreements or uncertainty were resolved after assessment of the full article by consensus between two reviewers or a third reviewer. For this review, chronic pain is defined as pain that lasts or recurs for three months or more, per International Association for the Study of Pain (IASP) guidelines and International Classification of Diseases (ICD-11).^17^

### Data extraction

Data were independently extracted by ZL and CL via a customized form specifying study design, study instrument, population, age range, data collection setting, urban/rural settings, number of subjects with response rate, sex ratio, definition of pain, and pain location. The second amendment to the protocol was that too few (2/27) studies reported extractable data on psychosocial predictors of chronic pain (such as history of depression or anxiety) for meaningful analysis to take place. Therefore, these data were not included.

### Statistical methods

Pain prevalence meta-analysis was conducted using the generalized linear mixed model (GLMM) with a logit transformation and a random effects model. We chose to use GLMM with logit transformation for the meta-analysis because the classic Freeman-Tukey double arsine transformation in meta-analysis has a back transformation that is dependent on a single “overall sample size”, which is chosen to represent the overall synthesized result.^18^ In the setting of a meta-analysis, this number is not well defined, and can be the arithmetic, geometric, or harmonic mean, or the inverse of the variances.^18^, ^19^, ^20^ When a study has a wide range of sample sizes (such as ours), the arithmetic, geometric, or harmonic mean, or the inverse of the variances can be very different. It is difficult to justify which of these numbers should be used as the “overall sample size” in the back transformation. Different “overall sample size” can lead to substantially different proportions calculated in the meta-analysis. In some cases, this can provide misleading conclusions, as demonstrated by the case study published by Schwazer et al.^20^ The GLMM, on other hand, does not suffer from this limitation and therefore was chosen for this analysis. A random effects model was used to control for inherent heterogeneity in our study population; therefore, it was assumed that heterogeneity in LMICs is constant over time and not associated with any individual variable. Subgroup analysis was performed by sex. Odds ratios by pain type were computed with a mixed-effects logistic regression model with random study effects to account for both fixed and random variables that may be associated with the heterogeneity inherent to data from LMICs. Ninety-five percent confidence intervals (CI) were computed with the Clopper-Pearson exact method. All statistical tests and forest plots were generated with R package meta version 4·18–1.^21^ Heterogeneity of studies, estimated as *I*^2^, represent the fraction of variability in study prevalence that is attributable to heterogeneity across studies rather than chance. A *P* value of ≤ 0·05 was considered statistically significant.

A quality assessment checklist for prevalence studies adapted from Hoy et al. and published by Nguyen et al. was used by two independent reviewers (ZL and CL) on included studies to assess for risk of bias within individual studies (Appendix 3).^22^^,^^23^ The checklist was previously published (accessible from https://doi.org/10.1371/journal.pone.0150970.s005). It includes nine items that rate the risk of bias as high (1 point) or low (0 point) in the following categories: representativeness of the sample, sampling technique, response rate, data collection method and tools, case definition, and statistical method. The sum of the nine-item scores is used to indicate low (0–3), moderate (4–6), or high (7–9) overall risk of study bias.

Sensitivity analyses were performed by either excluding all studies with moderate or high risk of bias or excluding studies that defined chronicity of pain as ≥6 months or a year. Funnel plots supplemented with Egger test of bias were performed to assess for possible publication bias.

### Role of the funding source

This study was funded by the Office of Medical Student Research, Vanderbilt University School of Medicine. It did not play a role in the writing of the manuscript or the decision to submit it for publication. . All authors had access to the data and agreed to submit the data for publication.

## Results

**Figure 1:**
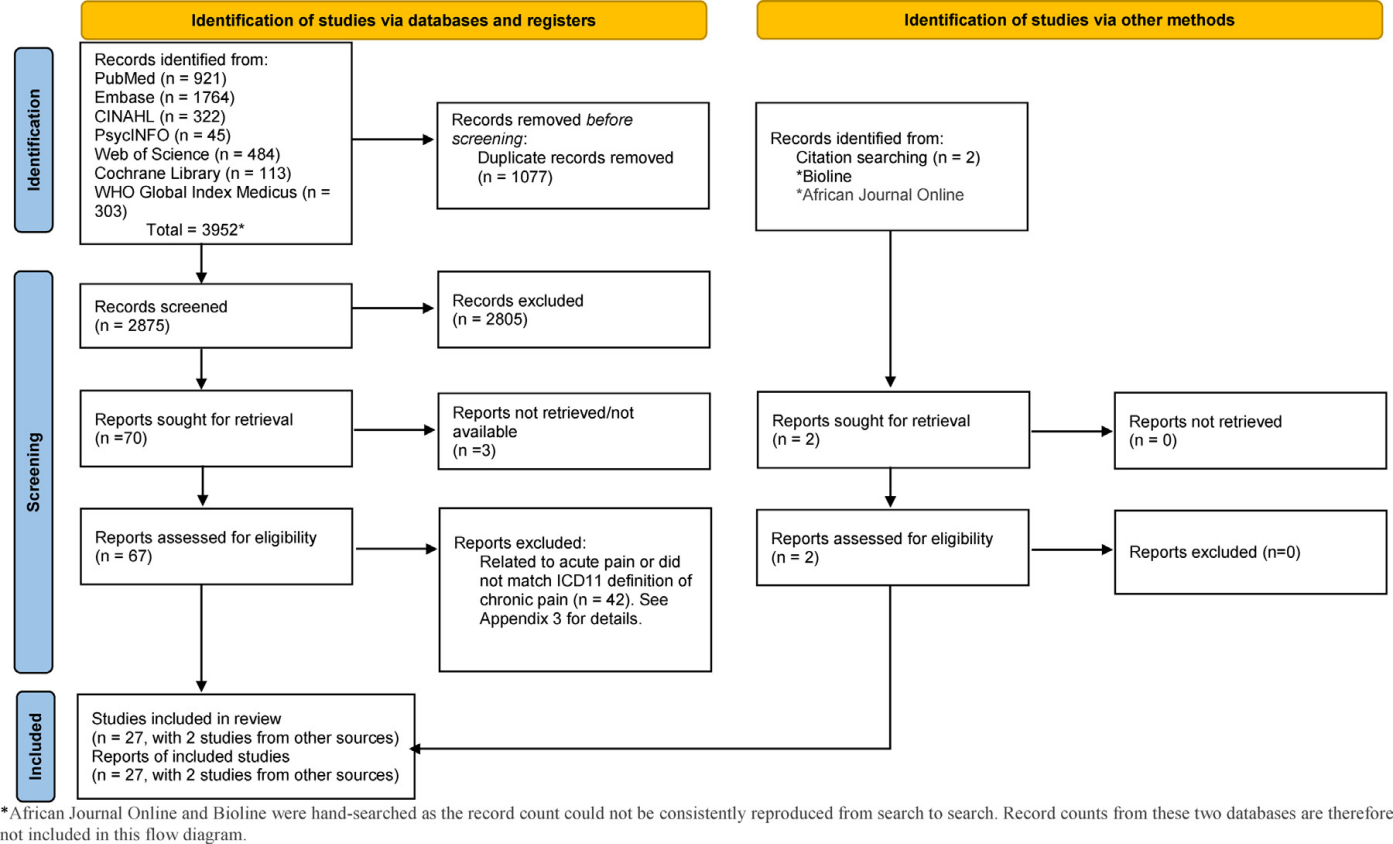
PRISMA 2020 flow diagram for new systematic reviews, including searches of databases and registries.

Our search identified 2875 articles. After abstract review, 70 were deemed potentially relevant (Figure 1). Of these, 45 were excluded: 3 did not have the full text available, 42 were not on paediatric chronic pain in LMICs (Appendix 2). Therefore, 27 studies (24 cross-sectional studies, two prospective studies, and one retrospective study) were included in the systematic review and meta-analysis (Table 1).^24^, ^25^, ^26^, ^27^, ^28^, ^29^, ^30^, ^31^, ^32^, ^33^, ^34^, ^35^, ^36^, ^37^, ^38^, ^39^, ^40^, ^41^, ^42^, ^43^, ^44^, ^45^, ^46^, ^47^, ^48^, ^49^, ^50^ The studies did not have overlapping samples. We found no study on chronic pain in neonates.

**Table 1:** Prevalence of chronic pain in children and adolescents (*N* represents the number of persons with chronic pain).

	Country	Year	Study Pop.	*N*	Study Details
Alimohammadi et al.^24^	Iran, Islamic Rep.	2016	1556	142	Age 6–13; population-based case-control study; at least three episodes of abdominal pain severe enough to affect daily activities, over a period of at least 3 months
Arruda et al.^25^	Brazil	2010	1994	17	Age 5–12; cross-sectional study; chronic migraine and tension-type headache (TTH) defined by ICHD2
Ayanniyi et al.^26^	Nigeria	2011	5671	35	Age 10–19; cross-sectional study; chronic pain>3 months
Bejia et al.^27^	Tunisia	2005	3185	669	Age 11–19; cross-sectional study; chronic pain >3 months
Boey et al.^28^	Malaysia	2000	622	50	Age 11–16; cross-sectional study; at least three episodes of abdominal pain severe enough to affect daily activities, over a period of at least 3 months
Boey et al.^29^	Malaysia	2003	456	1971	Age 12 only; cross-sectional study; at least three episodes of abdominal pain severe enough to affect daily activities, over a period of at least 3 months
Çagliyan Türk et al.^30^	Turkey	2020	476	35	Age 9–17; cross-sectional study; chronic pain >3 months
Chiwaridzo et al.^31^	Zimbabwe	2014	532	153	Age 13–19; cross-sectional study; pain recurrent within last year
Cruz et al.^32^	Ecuador	1985	1549	158	Age 0–19; cross-sectional study; recurrent and/or persistent severe headaches within the year preceding prevalence day
da Silva et al.^33^	Brazil	2010	579	14	Age 10–19; cross-sectional study; chronic headache defined by ICHD2
Devanarayana et al.^34^	Sri Lanka	2008	284	5	Age 5–15; cross-sectional study; at least three episodes of abdominal pain severe enough to affect daily activities, over a period of at least 3 months
Durmaz et al.^35^	Turkey	2013	734	77	Age 12–18; cross-sectional study; chronic pain >3 months
Franco- Micheloni et al.^36^	Brazil	2014	1109	61	Age 12–14; cross-sectional study; chronic pain >6 months
Gobina et al.^37^	Armenia, Albenia, TFYR Macedonia, Bulgaria, Republic of Moldova, Russian Federation, Ukraine	2019	92,544	7150	Age 11–15; cross-sectional study; chronic pain >6 months
Gupta et al.^38^	India	2009	2235	4	Age 12–19; cross-sectional study; chronic headache defined by ICHD2
Karli et al.^39^	Turkey	2006	2387	45	Age 12–17; cross-sectional study; chronic headache defined by ICHD2
Kumar et al.^40^	India	2017	1018	99	Age 5–16; cross-sectional study; chronic pain >3 months
Meziat Filho et al.^41^	Brazil	2015	990	184	Age 14–17; cross-sectional study; chronic pain >3 months
Meucci et al.^42^	Brazil	2018	1109	119	Age 13–19; cross-sectional study; chronic pain >3 months
Shaygan et al.^43^	Iran	2020	734	221	Age 12–19; cross-sectional study; chronic pain defined by ICD-11
Silva et al.^44^	Brazil	2011	1463	316	Age 7–11; longitudinal birth cohort study; the occurrence of pain or discomfort in the stomach in the last three months, strong enough to disrupt the child's daily activities such as playing, going to school or sleeping
Siu et al.^45^	China	2012	1518	463	Age 11–19; cross-sectional study; chronic pain >3 months
Ünalp et al.^46^	Turkey	2006	2384	1090	Age 14–18; cross-sectional study; chronic pain >3 months
Visudtibhan et al. ^47^	Thailand	2010	209	6	Age 11–13; 3-year prospective cohort study; chronic headache defined by ICHD2
Winkler et al.^48^	Tanzania	2009	2725	0	Age 0–10; cross-sectional study; chronic headache defined by ICHD2
Zapata et al.^49^	Brazil	2006	791	4	Age 10–18; cross-sectional study; chronic pain >3 months
Zhang et al.^50^	China	2007	8701	678	Age 6–15; cross-sectional study; pain recurrent within last year

ICHD2 = The International Classification of Headache Disorders, 2nd edition.

ICD11=International Classification of Diseases 11th Revision.

**Figure 2:**
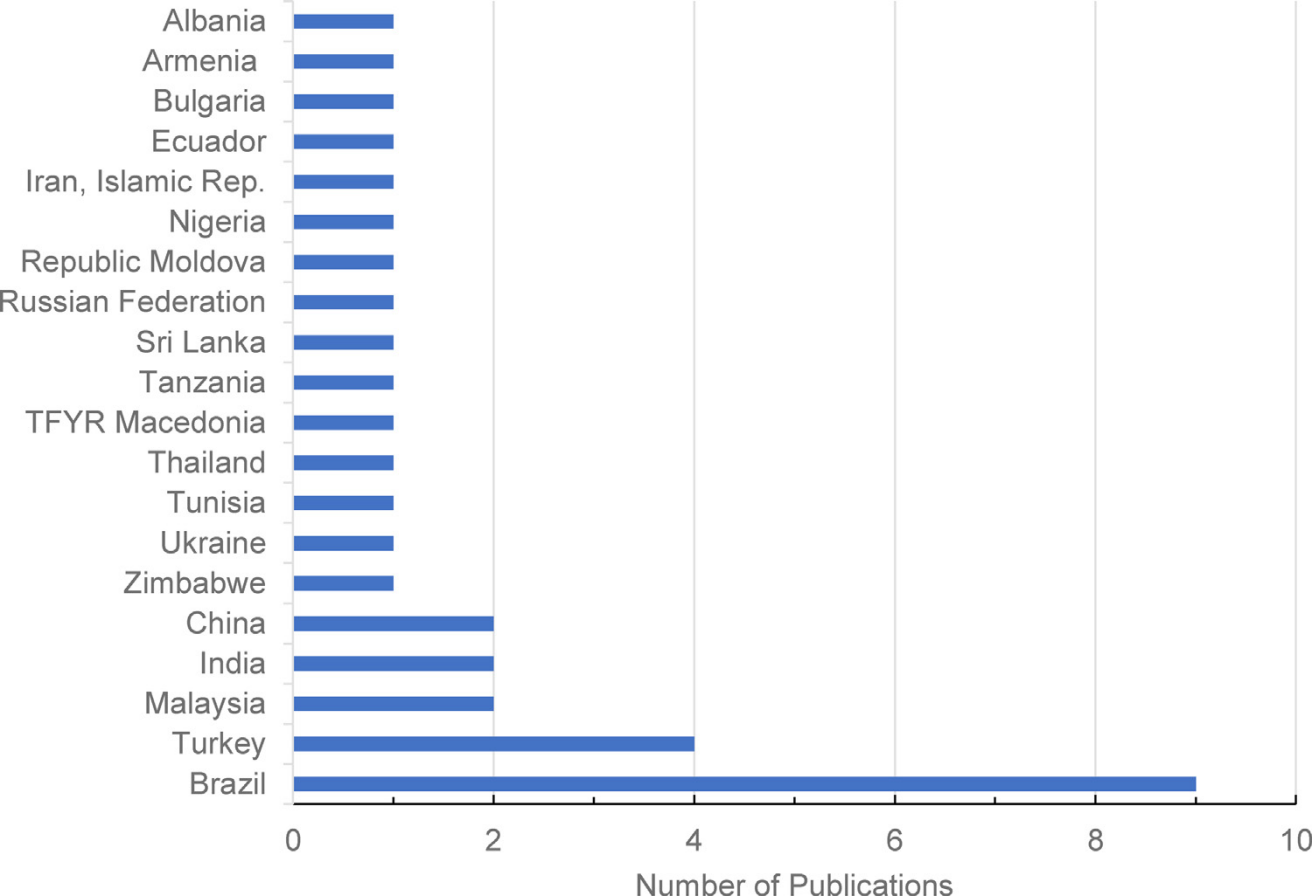
Number of publications per country. One study included multiple countries.

The included studies represented a total of 20 LMICs from six regions (Europe and Central Asia, Latin America and the Caribbean, Middle East and North Africa, East Asia and Pacific, South Asia, and Sub-Saharan Africa) (Figure 2). All studies except one reported on a single country.^37^ The multi-country study reported on multiple pain types in 42 countries, including seven LMICs (Armenia, Albania, TFYR Macedonia, Bulgaria, Republic of Moldova, Russian Federation, and Ukraine), whose data were extracted separately. Summary data from the LMICs were extracted and included in this review.

Twenty-one of 27 studies defined chronic pain as recurrent or persistent pain lasting for ≥3months, 3/27 as lasting 6 months or longer, and 3/27 as lasting for one year prior to study period. This is likely due to a change made by IASP in 2019 to define the duration of chronic pain as pain lasting ≥3 months; prior to this, it was defined as ≥3–6 months.^51^^,^^52^

Headaches were the most common study focus (*N* = 10), followed by musculoskeletal pain (*N* = 7), abdominal pain (*N* = 6), general/multi-site/any pain (*N* = 3), fibromyalgia (*N* = 3), and temporomandibular disorder (*N* = 1).

### Risk of bias

In all, >95% (26/27) of included studies have low overall risk of bias while the remainder one study had moderate risk of bias (Appendix 3). All included studies have a risk of bias in national representativeness and 6/27 in representing the target population. The response rates of the study range from 42 to ≥90% (median 95%), with five studies not reporting a response rate and 17/27 studies reporting a response rate of ≥75%.

Funnel plots with Egger tests were performed to assess possible publication bias (Appendix 4). There is evidence of publication bias for the Egger test when all studies were included (*P* < 0.05). However, when studies were sub-grouped by pain type (excluding TMJ as there is only one study), there is insufficient evidence of publication bias (all *P* ≥ 0.05 for the Egger tests), suggesting the publication bias observed when all studies were combined is attributable in part, to difference in pain type experienced by the study population.

### Paediatric chronic pain prevalence

**Figure 3:**
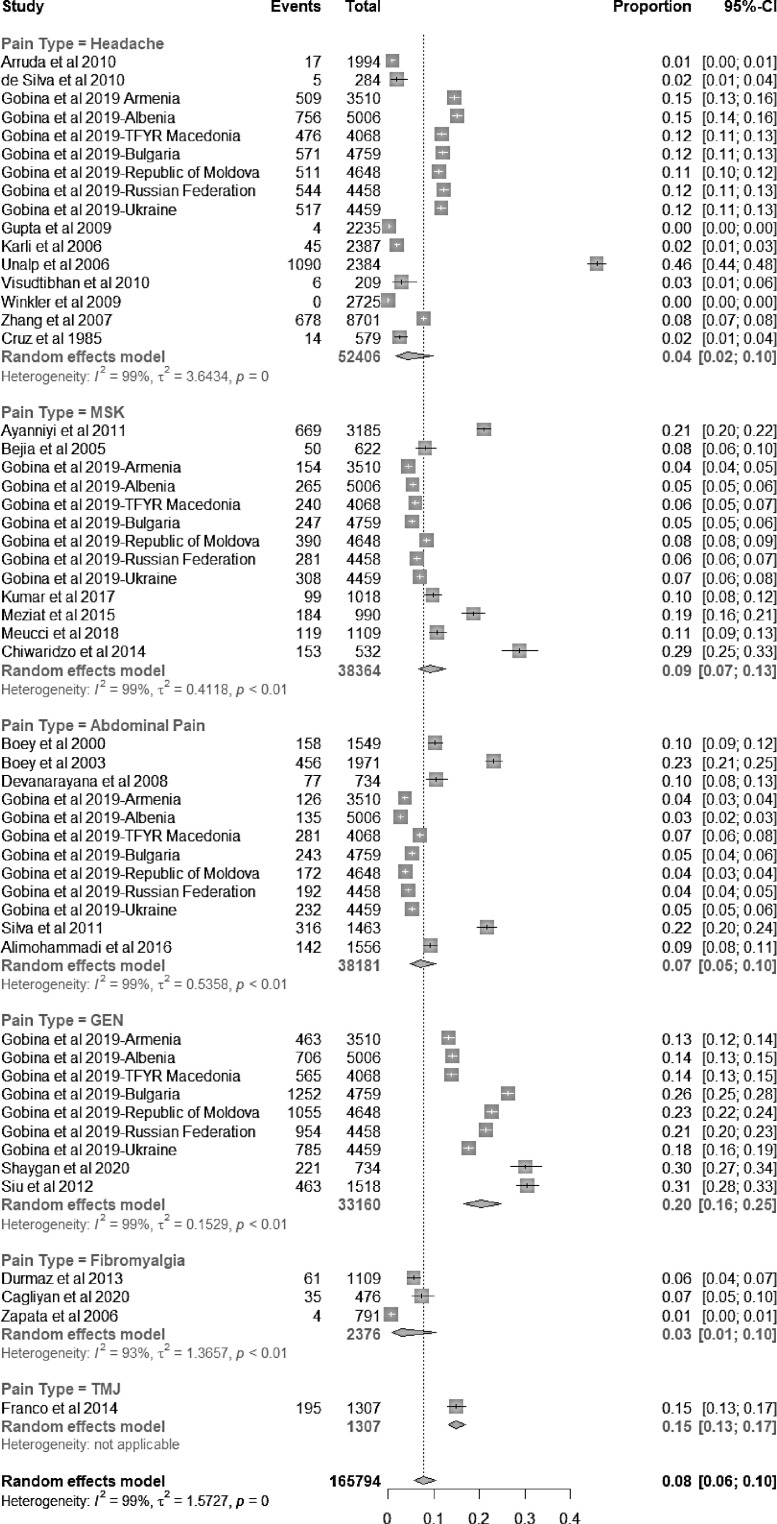
A forest plot of chronic pain prevalence estimates (proportion) with 95% confidence intervals (CI), excluding follow-up studies (random effects model, heterogeneity: *I*^2^=99%, *p* = 0). MSK: musculoskeletal pain. GEN: general or multi-site or any pain. TMJ: Temporomandibular joint (TMJ) syndrome. Gobina et al. is divided into countries and pain types as it is a multi-nation study that includes multiple pain types.

Figure 3 summarizes the prevalence estimates from the studies, stratified by pain type. Proportion ratios comparing subgroups by sex are included when available. The number of subjects included in each study ranged from 209 to 8701. A significantly high level of heterogeneity was found across all studies (*I^2^* >90%).

The prevalence of chronic pain in included studies has a pooled mean of 8% (95% CI 6–10). The prevalence rate for each type of chronic pain is estimated as follows: general/multi-site/any pain 20% (95% CI 16–25), MSK/back pain 9% (95% CI 7–13), abdominal pain 7% (95% CI 5–10), headache 4% (95% CI 2–10), and fibromyalgia 3% (95% CI 1–10). Only one study on chronic temporomandibular disorder pain met inclusion criteria, reporting a prevalence of 15%.^36^

A sensitivity analysis excluding studies of moderate or high risk of bias (*n* = 1, moderate bias; (prevalence: 7%, 95% CI 5–10, *I^2^* =97%) or considering studies with only consistent definition of the chronicity (only those with ≥3 months and not ≥6 months or 1 year) did not affect the conclusions of the meta-analysis (prevalence: 5%, 95% CI 2–11, *I^2^* =99%).

**Figure 4:**
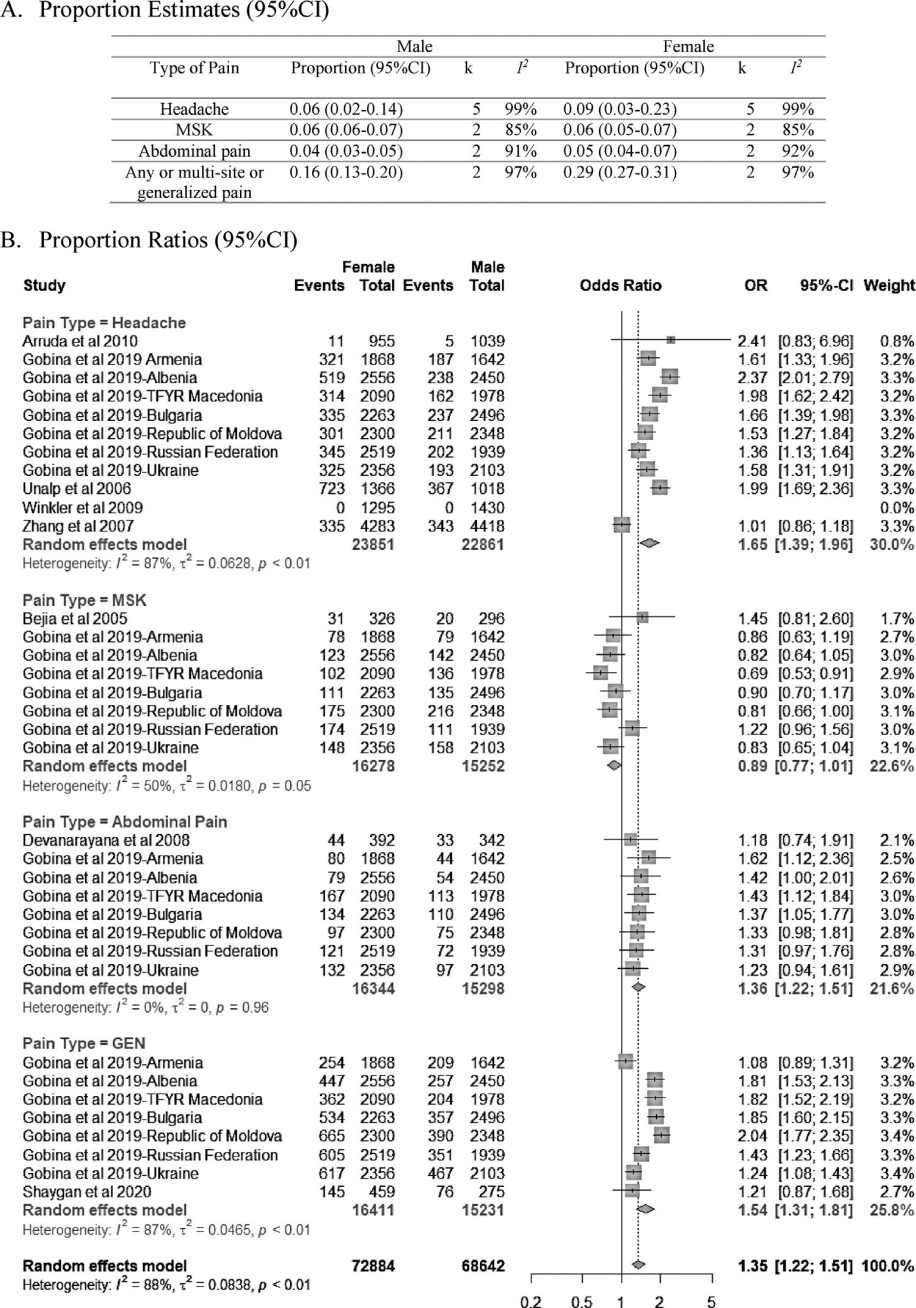
A and B. Proportion meta-analysis (random effects model, heterogeneity: *I*^2^ = 88%, *p* < 0.01). *k*= number of manuscripts; MSK = musculoskeletal pain; GEN = any/multi-site chronic pain; TMD = temporomandibular disorder.

In boys, chronic pain prevalence ranged from 0% to 36%. In girls, the prevalence of chronic pain ranged from 0% to 53%. Chronic headache, abdominal pain, and general/multi-site/any pain were, respectively, 1·65 (95% CI 1·39–1·96), 1·36 (95% CI 1·22–1·51), and 1·54 (95% CI 1·31–1·81) times more prevalent in girls compared with boys (Figure 4). The prevalence of musculoskeletal pain, however, was similar between subgroups (OR=0·89, 95% CI 0·77–1·01). Since only 1/27 study studied children <10 years exclusively and most of the studies (16/27) in this review used a minimal age cut-off of 10 years or higher, subgroup analysis by age was not possible.

## Discussion

This systematic review and meta-analysis examined the prevalence of paediatric chronic pain in LMICs. King et al. conducted a systematic review without meta-analysis on the epidemiology of paediatric chronic pain based largely on studies in high- and very-high–income countries, reporting prevalence rates of 8% to 83% for headache, 4% to 40% for musculoskeletal pain, 4% to 53% for abdominal pain, and 4% to 49% for generalized/multi-site pain.^7^ The range estimates reported in our studies are comparably lower and narrower: headache 0% to 46%, musculoskeletal pain 4% to 29%, abdominal pain 3% to 23%, generalized/multi-site pain 13% to 31%, and fibromyalgia 1% to 7%, likely because this review has inclusion criteria that included studies matching the current ICD-11 definition of chronic pain, which specifies a chronicity of ≥3 months, whereas King et al. included articles that reported chronic pain with unclear chronicity.

In the paediatric population, this review found a 4% (95% CI 2–10) prevalence of any chronic headache (chronic daily headache, chronic migraine, or chronic tension-type headache), which is comparable to the 5·5% migraine that was reported in LMICs in the Global Burden of Disease 2013 Study on Children and Adolescents.^53^

The Global Burden of Disease 2013 Study reported a 2% prevalence of paediatric low back and neck pain, whereas the Community Oriented Program for the Control of Rheumatic Diseases (COPCORD) estimated that MSK pain prevalence ranged from 14% to 47% in adults living in LMICs in Asia, Africa, and Central/South America, suggesting the prevalence of MSK pain in LMICs differs between paediatric and adult populations.^10^, ^54^, ^55^ In a meta-analysis on adults in LMICs, 25% of adults reported having MSK pain.^3^ In contrast, this review found 9% (95% CI 7–13) of children and adolescents in LMICs have chronic MSK pain.

Juvenile fibromyalgia syndrome and multi-site pain have overlapping characteristics, and both fall under the umbrella of generalized pain. In adults in LMICs, fibromyalgia was noted to have a prevalence of 6%, while widespread pain (excluding fibromyalgia) was 12%.^3^ In children living in a HIC, juvenile fibromyalgia syndrome was reported as 1% to 6%.^55^ In this review, the prevalence of juvenile fibromyalgia syndrome was comparable to that previously reported in adults in LMICs and children in HICs, at 3% (95% CI 1–10). Unlike adults, an estimate of 20% (95% CI 16–25) was noted for multi-site paediatric chronic pain (excluding fibromyalgia).

Recurrent abdominal pain is often considered to be functional in origin and accounts for up to 5% of annual family physician visits.^56^^,^^57^ A prior systematic review reported a 0·3% to 19% prevalence of recurrent abdominal pain in Western countries, whereas a study based in 42 countries (most of which were high-income) found an overall prevalence rate of chronic abdominal pain at 4·6%.^37^^,^^58^ Similarly, this review found that 6% (95% CI 4–9) of children and adolescents in LMICs had chronic abdominal pain.

In terms of sex differences, prevalence of headache, abdominal pain, and multi-site pain was higher in girls compared with boys, which is consistent with the prior review, although no sex difference was observed in MSK pain.^7^

A major limitation of this review is that most chronic pain studies published before 2019 varied widely in their definitions of chronic pain, if the term was defined at all. Although chronic pain has been considered as lasting ≥3 months in clinical practice since 1994, until 2019 when chronic pain arrived in ICD-11, it was often defined as ≥6 months in research.^17^^,^^52^ This limited the number of studies that could be included in this review. The narrowing in prevalence ranges in this review in comparison with the review by King et al. suggests that a rigorous enforcement of the chronicity definition of chronic pain has a strong impact on minimizing the heterogeneity observed in chronic pain data. For comparisons to be made between estimates of incidence and prevalence in different countries, it is essential that future studies adhere to the standard definition of chronic pain. Nevertheless, a significant amount of heterogeneity remains in our review, suggesting that the development of a standardized assessment tool for paediatric chronic pain is also indicated, as most studies included here were designed to study one type of chronic pain, rather than multiple subtypes of chronic pain. Our group has developed such a tool for adults in LMICs (the Vanderbilt Global Pain Survey, or VGPS), but to our knowledge no such tool exists for paediatric populations in LMICs.^59^^,^^60^

Although the included studies represent 20 LMICs, 61% of the studies came from Europe or Latin America, whereas Sub-Saharan Africa accounted for only 9%. There is a disproportionate number of studies from Brazil and Turkey, which are both classified as upper-middle income by the World Bank. Sixteen out of 20 LMICs included in this review are classified as having a high or very high Human Development Index (HDI) score. Since lower socioeconomic status has been found to be associated with higher pain prevalence, a future research focus on low-income countries may yield higher pain prevalences.^7^^,^^61^ There was also limited data from low-income countries and none from Central America. As a result, it is possible that this meta-analysis underestimates the prevalence of paediatric chronic pain in LMICs. Future studies should prioritize increasing the number of paediatric chronic pain studies in low-income countries and LMICs with low HDI scores.

This review is further limited by its dependence on self-reported and parent-reported data, which is subject to recall bias. Although selection bias was minimized by employing systematic sampling methods in cross-sectional studies, it cannot be excluded. Moreover, all included studies included a study population that is at risk of not being representative of the national paediatric population, either due to the use of an age cut-off or a focus on one region of the country. Future studies should consider expanding the age and regional limit of the studies to include all children and adolescents across an entire nation. Nevertheless, the majority of the studies in this review reported a response rate of >75%, and, therefore, the review has a low risk of attrition bias (Appendix 3). There is a paucity of paediatric chronic pain data from LMICs. In this review, the prevalence of paediatric chronic pain in LMICs is estimated to be at least 8% (95% CI 6–10). Existing studies suffer from significant heterogeneity in the assessment of chronic pain, which can confuse healthcare providers and policymakers and undermine the importance of treating chronic pain in children and adolescents. With the standardization of the definition of chronic pain in ICD-11, future studies should continue to develop culturally appropriate assessment tools to monitor incidence and prevalence of chronic pain, to lay the groundwork for tailoring treatment options to populations. The characterization of paediatric chronic pain burden in LMICs is an important first step in treating paediatric chronic pain and therefore must be prioritized.

## Contributors

ZL, HL, and CW contributed to study design. ZL, CL, HL, and CW contributed to data collection. ZL, CL, and CW contributed to data analysis. ZL and CL contributed to figure and table creation. All authors contributed to manuscript writing.

## Funding

This study is supported by the Office of Medical Student Research, Vanderbilt University School of Medicine. It did not play a role in the writing of the manuscript or the decision to submit it for publication.

## Data sharing statement

The dataset utilized in this study is available in Table 1 and are not subject to embargo or restrictions.

## Declaration of interests

The authors have no conflicts of interest to declare.

## Supplementary materials

Image, application 1

Image, application 2

Image, application 3

Image, application 4
