# Supplementary material for: Paediatric chronic pain prevalence in low- and middle-income countries: A systematic review and meta-analysis
Source: eClinicalMedicine. 2022 Feb 12;45:101296. doi: 10.1016/j.eclinm.2022.101296 (PMC8850335; doi:10.1016/j.eclinm.2022.101296)
Supplement: Supplementary file 1 [file mmc1.docx]

Appendix 1: Detailed search strategy for article selection (until January 7^th^, 2022)

**MEDLINE (via PubMed):** <http://www.library.vanderbilt.edu/eres?id=145>

**Date Last Searched: 1/7/2022**

("Chronic Pain"[Mesh] OR (("Pain"[Mesh] OR pain[tiab] OR pains[tiab] OR painful*[tiab] OR ache[tiab] OR aches[tiab] OR aching[tiab] OR backache[tiab] OR backaches[tiab] OR headache[tiab] OR headaches[tiab]) AND (chronic[tw] OR chronic*[tiab] OR persist* OR recurrent[tiab] OR recurring[tiab] OR reoccurrent[tiab] OR reoccurring[tiab]))) AND ("Developing Countries"[Mesh] OR LMIC[tiab] OR LMICs[tiab] OR LIC[tiab] OR LICs[tiab] OR MIC[tiab] OR MICs[tiab] OR low income country[tiab] OR low income countries[tiab] OR low income nation[tiab] OR low income nations[tiab] OR low income setting[tiab] OR low income settings[tiab] OR low income economy[tiab] OR low income economies[tiab] OR lower income country[tiab] OR lower income countries[tiab] OR lower income nations[tiab] OR lower income economies[tiab] OR middle income country[tiab] OR middle income countries[tiab] OR middle income nation[tiab] OR middle income nations[tiab] OR middle income setting[tiab] OR middle income settings[tiab] OR middle income economy[tiab] OR middle income economies[tiab] OR developing country[tiab] OR developing countries[tiab] OR developing nation[tiab] OR developing nations[tiab] OR developing economy[tiab] OR developing economies[tiab] OR under developed country[tiab] OR under developed countries[tiab] OR under developed nation[tiab] OR under developed nations[tiab] OR less developed country[tiab] OR less developed countries[tiab] OR less developed nation[tiab] OR less developed nations[tiab] OR less resourced country[tiab] OR less resourced countries[tiab] OR low resource country[tiab] OR low resource countries[tiab] OR low resource nations[tiab] OR low resource setting[tiab] OR low resource settings[tiab] OR low resourced country[tiab] OR low resourced countries[tiab] OR low resourced setting[tiab] OR low resourced settings[tiab] OR third world country[tiab] OR third world countries[tiab] OR third world nation[tiab] OR third world nations[tiab] OR "Africa"[Mesh] OR Africa[tiab] OR "Asia"[Mesh] OR Asia[tiab] OR "Central America"[Mesh] OR Central America[tiab] OR "Latin America"[Mesh] OR Latin America[tiab] OR "South America"[Mesh] OR South America[tiab] OR "Afghanistan"[Mesh] OR Afghanistan[tiab] OR "Albania"[Mesh] OR Albania[tiab] OR Albanian[tiab] OR "Algeria"[Mesh] OR Algeria[tiab] OR Algerian[tiab] OR "Angola"[Mesh] OR Angola[tiab] OR Angolan[tiab] OR "Argentina"[Mesh] OR Argentina[tiab] OR Argentinian[tiab] OR "Armenia"[Mesh] OR Armenia[tiab] OR Armenian[tiab] OR "Azerbaijan"[Mesh] OR Azerbaijan[tiab] OR "Bangladesh"[Mesh] OR Bangladesh[tiab] OR "Republic of Belarus"[Mesh] OR Belarus[tiab] OR Byelarus[tiab] OR Belorussia[tiab] OR Belorussian[tiab] OR Byelorussia[tiab] OR Byelorussian[tiab] OR "Belize"[Mesh] OR Belize[tiab] OR British Honduras[tiab] OR "Benin"[Mesh] OR Benin[tiab] OR "Bhutan"[Mesh] OR Bhutan[tiab] OR "Bolivia"[Mesh] OR Bolivia[tiab] OR Bolivian[tiab] OR "Bosnia and Herzegovina"[Mesh] OR Bosnia[tiab] OR Bosnian[tiab] OR Herzegovina[tiab] OR Hercegovina[tiab] OR Bosnia-Herzegovina[tiab] OR "Botswana"[Mesh] OR Botswana[tiab] OR Bechuanaland[tiab] OR "Brazil"[Mesh] OR Brazil[tiab] OR Brazilian[tiab] OR "Bulgaria"[Mesh] OR Bulgaria[tiab] OR Bulgarian[tiab] OR "Burkina Faso"[Mesh] OR Burkina Faso[tiab] OR "Burundi"[Mesh] OR Burundi[tiab] OR Urundi[tiab] OR "Cabo Verde"[Mesh] OR Cabo Verde[tiab] OR Cape Verde[tiab] OR "Cambodia"[Mesh] OR Cambodia[tiab] OR Cambodian[tiab] OR "Cameroon"[Mesh] OR Cameroon[tiab] OR "Central African Republic"[Mesh] OR Central African Republic[tiab] OR "Chad"[Mesh] OR Chad[tiab] OR Chadian[tiab] OR "China"[Mesh] OR China[tiab] OR Chinese[tiab] OR "Colombia"[Mesh] OR Colombia[tiab] OR Colombian[tiab] OR "Comoros"[Mesh] OR Comoros[tiab] OR Comoro Islands[tiab] OR "Congo"[Mesh] OR Congo[tiab] OR "Costa Rica"[Mesh] OR Costa Rica[tiab] OR Costa Rican[tiab] OR "Cote d'Ivoire"[Mesh] OR Cote d'Ivoire[tiab] OR Ivory Coast[tiab] OR "Cuba"[Mesh] OR Cuba[tiab] OR Cuban[tiab] OR "Djibouti"[Mesh] OR Djibouti[tiab] OR "Dominica"[Mesh] OR Dominica[tiab] OR Dominican[tiab] OR "Dominican Republic"[Mesh] OR Dominican Republic[tiab] OR "Ecuador"[Mesh] OR Ecuador[tiab] OR "Egypt"[Mesh] OR Egypt[tiab] OR Egyptian[tiab] OR "El Salvador"[Mesh] OR El Salvador[tiab] OR Salvadoran[tiab] OR "Equatorial Guinea"[Mesh] OR Equatorial Guinea[tiab] OR "Eritrea"[Mesh] OR Eritrea[tiab] OR Eritrean[tiab] OR "Eswatini"[Mesh] OR Eswatini[tiab] OR "Ethiopia"[Mesh] OR Ethiopia[tiab] OR Ethiopian[tiab] OR "Fiji"[Mesh] OR Fiji[tiab] OR "Gabon"[Mesh] OR Gabon[tiab] OR Gabonese[tiab] OR "Gambia"[Mesh] OR Gambia[tiab] OR Gambian[tiab] OR "Georgia (Republic)"[Mesh] OR Georgia[tiab] OR Georgian[tiab] OR "Ghana"[Mesh] OR Ghana[tiab] OR "Grenada"[Mesh] OR Grenada[tiab] OR "Guatemala"[Mesh] OR Guatemala[tiab] OR Guatemalan[tiab] OR "Guinea"[Mesh] OR Guinea[tiab] OR Guinean[tiab] OR "Guinea-Bissau"[Mesh] OR Guinea-Bissau[tiab] OR "Guyana"[Mesh] OR Guyana[tiab] OR "Haiti"[Mesh] OR Haiti[tiab] OR Haitian[tiab] OR "Honduras"[Mesh] OR Honduras[tiab] OR "India"[Mesh] OR India[tiab] OR Indian[tiab] OR "Indonesia"[Mesh] OR Indonesia[tiab] OR Indonesian[tiab] OR "Iran"[Mesh] OR Iran[tiab] OR "Iraq"[Mesh] OR Iraq[tiab] OR "Jamaica"[Mesh] OR Jamaica[tiab] OR Jamaican[tiab] OR "Jordan"[Mesh] OR Jordan[tiab] OR "Kazakhstan"[Mesh] OR Kazakhstan[tiab] OR "Kenya"[Mesh] OR Kenya[tiab] OR Kenyan[tiab] OR Kiribati[tiab] OR "Kosovo"[Mesh] OR Kosovo[tiab] OR "Kyrgyzstan"[Mesh] OR Kyrgyzstan[tiab] OR "Laos"[Mesh] OR Laos[tiab] OR Lao PDR[tiab] OR "Lao People’s Democratic Republic"[tiab] OR "Lebanon"[Mesh] OR Lebanon[tiab] OR Lebanese[tiab] OR "Lesotho"[Mesh] OR Lesotho[tiab] OR "Liberia"[Mesh] OR Liberia[tiab] OR Liberian[tiab] OR "Libya"[Mesh] OR Libya[tiab] OR Libyan[tiab] OR "Madagascar"[Mesh] OR Madagascar[tiab] OR "Malawi"[Mesh] OR Malawi[tiab] OR "Malaysia"[Mesh] OR Malaysia[tiab] OR Malaysian[tiab] OR "Indian Ocean Islands"[Mesh] OR Maldives[tiab] OR "Mali"[Mesh] OR Mali[tiab] OR "Micronesia"[Mesh] OR Micronesia[tiab] OR Micronesian[tiab] OR Marshall Islands[tiab] OR "Mauritania"[Mesh] OR Mauritania[tiab] OR Mauritanian[tiab] OR "Mexico"[Mesh] OR Mexico[tiab] OR "Moldova"[Mesh] OR Moldova[tiab] OR "Mongolia"[Mesh] OR Mongolia[tiab] OR Mongolian[tiab] OR "Montenegro"[Mesh] OR Montenegro[tiab] OR "Morocco"[Mesh] OR Morocco[tiab] OR Moroccan[tiab] OR "Mozambique"[Mesh] OR Mozambique[tiab] OR "Myanmar"[Mesh] OR Myanmar[tiab] OR "Namibia"[Mesh] OR Namibia[tiab] OR Namibian[tiab] OR "Nepal"[Mesh] OR Nepal[tiab] OR "Nicaragua"[Mesh] OR Nicaragua[tiab] OR Nicaraguan[tiab] OR "Niger"[Mesh] OR Niger[tiab] OR "Nigeria"[Mesh] OR Nigeria[tiab] OR Nigerian[tiab] OR "Republic of North Macedonia"[Mesh] OR North Macedonia[tiab] OR "Pakistan"[Mesh] OR Pakistan[tiab] OR Pakistani[tiab] OR "Papua New Guinea"[Mesh] OR Papua New Guinea[tiab] OR "Paraguay"[Mesh] OR Paraguay[tiab] OR "Peru"[Mesh] OR Peru[tiab] OR "Philippines"[Mesh] OR Philippines[tiab] OR Philippine[tiab] OR Philippino[tiab] OR "Russia"[Mesh] OR Russia[tiab] OR Russian[tiab] OR "Rwanda"[Mesh] OR Rwanda[tiab] OR Rwandan[tiab] OR "Samoa"[Mesh] OR Samoa[tiab] OR Samoan[tiab] OR Samoan Islands[tiab] OR "Sao Tome and Principe"[Mesh] OR Sao Tome and Principe[tiab] OR "Senegal"[Mesh] OR Senegal[tiab] OR "Serbia"[Mesh] OR Serbia[tiab] OR Serbian[tiab] OR "Sierra Leone"[Mesh] OR Sierra Leone[tiab] OR "Melanesia"[Mesh] OR Melanesia[tiab] OR Melanesian[tiab] OR Solomon Islands[tiab] OR "Somalia"[Mesh] OR Somalia[tiab] OR Somali[tiab] OR "South Africa"[Mesh] OR South Africa[tiab] OR South African[tiab] OR "South Sudan"[Mesh] OR South Sudan[tiab] OR "Sri Lanka"[Mesh] OR Sri Lanka[tiab] OR Sri Lankan[tiab] OR "Saint Lucia"[Mesh] OR Saint Lucia[tiab] OR St Lucia[tiab] OR St. Lucia[tiab] OR "Saint Vincent and the Grenadines"[Mesh] OR Grenadines[tiab] OR "Sudan"[Mesh] OR Sudan[tiab] OR Sudanese[tiab] OR "Suriname"[Mesh] OR Suriname[tiab] OR "Syria"[Mesh] OR Syria[tiab] OR Syrian[tiab] OR "Tajikistan"[Mesh] OR Tajikistan[tiab] OR Tadjikistan[tiab] OR Tadzhikistan[tiab] OR Tadzhik[tiab] OR "Tanzania"[Mesh] OR Tanzania[tiab] OR Tanzanian[tiab] OR "Thailand"[Mesh] OR Thailand[tiab] OR "Timor-Leste"[Mesh] OR Timor-Leste[tiab] OR "Togo"[Mesh] OR Togo[tiab] OR "Tonga"[Mesh] OR Tonga[tiab] OR "Tunisia"[Mesh] OR Tunisia[tiab] OR "Turkey"[Mesh] OR Turkey[tiab] OR Turkish[tiab] OR "Turkmenistan"[Mesh] OR Turkmenistan[tiab] OR Tuvalu[tiab] OR "Uganda"[Mesh] OR Uganda[tiab] OR Ugandan[tiab] OR "Ukraine"[Mesh] OR Ukraine[tiab] OR Ukrainian[tiab] OR "Uzbekistan"[Mesh] OR Uzbekistan[tiab] OR Uzbek[tiab] OR "Vanuatu"[Mesh] OR Vanuatu[tiab] OR "Vietnam"[Mesh] OR Vietnam[tiab] OR Viet Nam[tiab] OR Vietnamese[tiab] OR "Middle East"[Mesh] OR Middle East[tiab] OR Middle Eastern[tiab] OR West Bank[tiab] OR Gaza[tiab] OR "Yemen"[Mesh] OR Yemen[tiab] OR "Zambia"[Mesh] OR Zambia[tiab] OR Zambian[tiab] OR "Zimbabwe"[Mesh] OR Zimbabwe[tiab]) AND ("Prevalence"[Mesh] OR prevalen*[tiab] OR "Incidence"[Mesh] OR incidence[tiab] OR incidences[tiab] OR "Epidemiology"[Mesh] OR epidemiolog*[tiab]) AND ("Pediatrics"[Mesh] OR pediatric[tiab] OR pediatrics[tiab] OR paediatric[tiab] OR paediatrics[tiab] OR "Child"[Mesh] OR children*[tiab] OR child[tiab] OR childs[tiab] OR child’s[tiab] OR childhood[tiab] OR "Infant"[Mesh] OR infant[tiab] OR infants[tiab] OR infantile[tiab] OR infancy[tiab] OR newborn[tiab] OR newborns[tiab] OR neonat*[tiab] OR baby[tiab] OR babies[tiab] OR toddler[tiab] OR toddlers[tiab] OR preschool*[tiab] OR pre-school*[tiab] OR school age*[tiab] OR schoolchildren[tiab] OR "Adolescent"[Mesh] OR teen*[tiab] OR youth[tiab] OR youths[tiab] OR juvenile[tiab] OR juveniles[tiab] OR puberty[tiab] OR pubescent[tiab] OR adolescen*[tiab] OR preadolescen*[tiab] OR pre-adolescen*[tiab] OR boy[tiab] OR boys[tiab] OR girl[tiab] OR girls[tiab]) AND Humans[Filter] AND 1883/01/01:2020/12/01[dp] NOT ("Review"[Publication Type] OR "Case Reports"[Publication Type] OR "Meeting Abstract"[Publication Type])

**921 Results**

Excluded Reviews, Case Reports, and Conference/Meeting Abstracts

Limited to publication dates 1/7/2022 and prior

Limited to Humans

**Embase (OvidSP):** <https://ckm.vumc.org/ckm/diglib/ckmres.html?rid=197>

**Date Last Searched: 1/7/2022**

((exp chronic pain/ or ((exp pain/ or (pain or pains or painful* or ache or aches or aching or backache or backaches or headache or headaches).ab,kw,ti.) and (chronic.ab,hw,kw,ti. or (chronic* or persist* or recurrent or recurring or reoccurrent or reoccurring).ab,kw,ti.))) and (exp low income country/ or exp middle income country/ or exp developing country/ or exp Africa/ or African/ or exp Asia/ or Asian/ or exp Central America/ or Central American/ or exp "South and Central America"/ or exp South America/ or South American/ or (LMIC or LMICs or LIC or LICs or MIC or MICs or low income country or low income countries or low income nation or low income nations or low income setting or low income settings or low income economy or low income economies or lower income country or lower income countries or lower income nation or lower income nations or lower income economy or lower income economies or middle income country or middle income countries or middle income nation or middle income nations or middle income setting or middle income settings or middle income economy or middle income economies or developing country or developing countries or developing nation or developing nations or developing economy or developing economies or under developed country or under developed countries or under developed nation or under developed nations or less developed country or less developed countries or less developed nation or less developed nations or less resourced country or less resourced countries or less resourced nation or less resourced nations or low resource country or low resource countries or low resource nation or low resource nations or low resource setting or low resource settings or low resourced country or low resourced countries or low resourced nation or low resourced nations or low resourced setting or low resourced settings or third world country or third world countries or third world nation or third world nations or Africa or Asia or Central America or Latin America or South America).ab,kw,ti. or exp Afghanistan/ or exp Albania/ or exp "Albanian (people)"/ or exp "Albanian (citizen)"/ or exp Algeria/ or exp Algerian/ or exp Angola/ or exp Angolan/ or exp Argentina/ or exp Argentinian/ or exp Armenia/ or exp "Armenian (people)"/ or exp "Armenian (citizen)"/ or exp Azerbaijan/ or exp Bangladesh/ or exp Belarus/ or exp Belize/ or exp Benin/ or exp Bhutan/ or exp Bolivia/ or exp Bolivian/ or exp "Bosnia and Herzegovina"/ or exp "Bosnian (citizen)"/ or exp Botswana/ or exp Brazil/ or exp Brazilian/ or exp Bulgaria/ or exp "Bulgarian (people)"/ or exp "Bulgarian (citizen)"/ or exp Burkina Faso/ or exp Burundi/ or exp Cape Verde/ or exp Cambodia/ or exp Cambodian/ or exp Cameroon/ or exp Central African Republic/ or exp Chad/ or exp "Chadian (citizen)"/ or exp China/ or exp Chinese/ or exp Colombia/ or exp Colombian/ or exp Comoros/ or exp Congo/ or exp Costa Rica/ or exp Costa Rican/ or exp Cote d'Ivoire/ or exp Cuba/ or exp Cuban/ or exp Djibouti/ or exp Dominica/ or exp "Dominican (Dominican Republic)"/ or exp "Dominican (Dominica)"/ or exp Dominican Republic/ or exp Ecuador/ or exp Egypt/ or exp Egyptian/ or exp El Salvador/ or exp Salvadoran/ or exp Equatorial Guinea/ or exp Eritrea/ or exp Eritrean/ or exp Eswatini/ or exp Ethiopia/ or exp Ethiopian/ or exp Fiji/ or exp Gabon/ or exp Gabonese/ or exp Gambia/ or exp Gambian/ or exp "Georgia (republic)"/ or exp "Georgian (citizen)"/ or exp Ghana/ or exp Grenada/ or exp Guatemala/ or exp Guatemalan/ or exp Guinea/ or exp Guinean/ or exp Guinea-Bissau/ or exp Guyana/ or exp Haiti/ or exp Haitian/ or exp Honduras/ or exp India/ or exp Indian/ or exp Indonesia/ or exp Indonesian/ or exp Iran/ or exp Iraq/ or exp Jamaica/ or exp Jamaican/ or exp Jordan/ or exp Kazakhstan/ or exp Kenya/ or exp Kenyan/ or exp Kiribati/ or exp Kosovo/ or exp Kyrgyzstan/ or exp Laos/ or exp Lebanon/ or exp Lebanese/ or exp Lesotho/ or exp Liberia/ or exp Liberian/ or exp Libyan Arab Jamahiriya/ or exp Libyan/ or exp Madagascar/ or exp Malawi/ or exp Malaysia/ or exp Malaysian/ or exp Indian Ocean/ or exp Maldives/ or exp Mali/ or exp Marshall Islands/ or exp Mauritania/ or exp Mauritanian/ or exp Mexico/ or exp Mexican/ or exp "Federated States of Micronesia"/ or exp Micronesian/ or exp Moldova/ or exp Mongolia/ or exp "Mongolian (citizen)"/ or exp "Montenegro (republic)"/ or exp Morocco/ or exp Moroccan/ or exp Mozambique/ or exp Myanmar/ or exp Namibia/ or exp Namibian/ or exp Nepal/ or exp Nicaragua/ or exp Nicaraguan/ or exp Niger/ or exp Nigeria/ or exp Nigerian/ or exp "Republic of North Macedonia"/ or exp Pakistan/ or exp Pakistani/ or exp Papua New Guinea/ or exp Paraguay/ or exp Peru/ or exp Philippines/ or exp Russian Federation/ or exp "Russian (citizen)"/ or exp Rwanda/ or exp Rwandan/ or exp Samoa/ or exp "Samoan (people)"/ or exp Samoan Islands/ or exp "Sao Tome and Principe"/ or exp Senegal/ or exp Serbia/ or exp "Serbian (citizen)"/ or exp Sierra Leone/ or exp Melanesia/ or exp Melanesian/ or exp Solomon Islands/ or exp Somalia/ or exp "Somali (citizen)"/ or exp South Africa/ or exp South African/ or exp South Sudan/ or exp Sri Lanka/ or exp Sri Lankan/ or exp Saint Lucia/ or exp "Saint Vincent and the Grenadines"/ or exp Sudan/ or exp Sudanese/ or exp Suriname/ or exp Syrian Arab Republic/ or exp Syrian/ or exp Tajikistan/ or exp Tanzania/ or exp Tanzanian/ or exp Thailand/ or exp Timor-Leste/ or exp Togo/ or exp "Tonga (people)"/ or exp Tonga/ or exp Tunisia/ or exp "Turkey (republic)"/ or exp Turkish citizen/ or exp Turkmenistan/ or exp Tuvalu/ or exp Uganda/ or exp Ugandan/ or exp Ukraine/ or exp Uzbekistan/ or exp "Uzbek (people)"/ or exp "Uzbek (citizen)"/ or exp Vanuatu/ or exp "Ukrainian (people)"/ or exp "Ukrainian (citizen)"/ or exp Viet Nam/ or exp Vietnamese/ or exp Middle East/ or exp Yemen/ or exp Zambia/ or exp Zambian/ or exp Zimbabwe/ or (Afghanistan or Albania or Albanian or Algeria or Algerian or Angola or Angolan or Argentina or Argentinian or Armenia or Armenian or Azerbaijan or Bangladesh or Belarus or Byelarus or Belorussia or Belorussian or Byelorussia or Byelorussian or Belize or British Honduras or Benin or Bhutan or Bolivia or Bolivian or Bosnia or Bosnian or Herzegovina or Hercegovina or Bosnia-Herzegovina or Botswana or Bechuanaland or Brazil or Brazilian or Bulgaria or Bulgarian or Burkina Faso or Burundi or Urundi or Cabo Verde or Cape Verde or Cambodia or Cambodian or Cameroon or Central African Republic or Chad or Chadian or China or Chinese or Colombia or Colombian or Comoros or Comoro Islands or Congo or Costa Rica or Costa Rican or Cote d'Ivoire or Ivory Coast or Cuba or Cuban or Djibouti or Dominica or Dominican or Dominican Republic or Ecuador or Egypt or Egyptian or El Salvador or Salvadoran or Equatorial Guinea or Eritrea or Eritrean or Eswatini or Ethiopia or Ethiopian or Fiji or Gabon or Gabonese or Gambia or Gambian or Georgia or Georgian or Ghana or Grenada or Guatemala or Guatemalan or Guinea or Guinean or Guinea-Bissau or Guyana or Haiti or Haitian or Honduras or India or Indian or Indonesia or Indonesian or Iran or Iraq or Jamaica or Jamaican or Jordan or Kazakhstan or Kenya or Kenyan or Kiribati or Kosovo or Kyrgyzstan or Laos or Lao PDR or "Lao People's Democratic Republic" or Lebanon or Lebanese or Lesotho or Liberia or Liberian or Libya or Libyan or Madagascar or Malawi or Malaysia or Malaysian or Maldives or Mali or Micronesia or Micronesian or Marshall Islands or Mauritania or Mauritanian or Mexico or Moldova or Mongolia or Mongolian or Montenegro or Morocco or Moroccan or Mozambique or Myanmar or Namibia or Namibian or Nepal or Nicaragua or Nicaraguan or Niger or Nigeria or Nigerian or North Macedonia or Pakistan or Pakistani or Papua New Guinea or Paraguay or Peru or Philippines or Philippine or Philippino or Russia or Russian or Rwanda or Rwandan or Samoa or Samoan or Samoan Islands or "Sao Tome and Principe" or Senegal or Serbia or Serbian or Sierra Leone or Melanesia or Melanesian or Solomon Islands or Somalia or Somali or South Africa or South African or South Sudan or Sri Lanka or Sri Lankan or Grenadines or Sudan or Sudanese or Suriname or Syria or Syrian or Tajikistan or Tadjikistan or Tadzhikistan or Tadzhik or Tanzania or Tanzanian or Thailand or Timor-Leste or Togo or Tonga or Tunisia or Turkey or Turkish or Turkmenistan or Tuvalu or Uganda or Ugandan or Ukraine or Ukrainian or Uzbekistan or Uzbek or Vanuatu or Vietnam or Viet Nam or Vietnamese or Middle East or Middle Eastern or West Bank or Gaza or Yemen or Zambia or Zambian or Zimbabwe).ab,kw,ti.) and (exp prevalence/ or exp incidence/ or exp epidemiology/ or (prevalen* or incidence or incidences or epidemiolog*).ab,kw,ti.) and (exp infant/ or exp child/ or exp adolescent/ or exp adolescence/ or exp pediatrics/ or (pediatric or pediatrics or paediatric or paediatrics or children* or child or childs or child's or childhood or infant or infants or infantile or infancy or newborn or newborns or neonat* or baby or babies or toddler or toddlers or preschool* or pre-school* or school age* or schoolchildren or teen* or youth or youths or juvenile or juveniles or puberty or pubescent or adolescen* or preadolescen* or pre-adolescen* or boy or boys or girl or girls).ab,kw,ti.) and exp human/) not ((review or conference abstract).pt. or exp case study/ or exp case report/)

Excluded Reviews, Case Studies, Case Reports, and Conference/Meeting Abstracts

Limited to Humans

**1764 Results:** Limit to publication dates 2022 and prior (cannot limit by month/day)

**CINAHL (EBSCOhost):** <http://www.library.vanderbilt.edu/eres?id=29>

**Date Last Searched: 1/7/2022**

(MH "Chronic Pain" OR ((MH "Pain+" OR TI (pain OR pains OR painful* OR ache OR aches OR aching OR backache OR backaches OR headache OR headaches) OR AB (pain OR pains OR painful* OR ache OR aches OR aching OR backache OR backaches OR headache OR headaches)) AND (MW chronic OR TI (chronic* OR persist* OR recurrent OR recurring OR reoccurrent OR reoccurring) OR AB (chronic* OR persist* OR recurrent OR recurring OR reoccurrent OR reoccurring)))) AND (MH “Low and Middle Income Countries” OR MH "Developing Countries" OR MH “Africa+” OR MH “Asia+” OR MH “Central America+” OR MH “Latin America” OR MH “South America+” OR TI (LMIC OR LMICs OR LIC OR LICs OR MIC OR MICs OR low income country OR low income countries OR low income nation OR low income nations OR low income setting OR low income settings OR low income economy OR low income economies OR lower income country OR lower income countries OR lower income nation OR lower income nations OR lower income economy OR lower income economies OR middle income country OR middle income countries OR middle income nation OR middle income nations OR middle income setting OR middle income settings OR middle income economy OR middle income economies OR developing country OR developing countries OR developing nation OR developing nations OR developing economy OR developing economies OR under developed country OR under developed countries OR under developed nation OR under developed nations OR less developed country OR less developed countries OR less developed nation OR less developed nations OR less resourced country OR less resourced countries OR less resourced nation OR less resourced nations OR low resource country OR low resource countries OR low resource nation OR low resource nations OR low resource setting OR low resource settings OR low resourced country OR low resourced countries OR low resourced nation OR low resourced nations OR low resourced setting OR low resourced settings OR third world country OR third world countries OR third world nation OR third world nations OR Africa OR Asia OR Central America OR Latin America OR South America) OR AB (LMIC OR LMICs OR LIC OR LICs OR MIC OR MICs OR low income country OR low income countries OR low income nation OR low income nations OR low income setting OR low income settings OR low income economy OR low income economies OR lower income country OR lower income countries OR lower income nation OR lower income nations OR lower income economy OR lower income economies OR middle income country OR middle income countries OR middle income nation OR middle income nations OR middle income setting OR middle income settings OR middle income economy OR middle income economies OR developing country OR developing countries OR developing nation OR developing nations OR developing economy OR developing economies OR under developed country OR under developed countries OR under developed nation OR under developed nations OR less developed country OR less developed countries OR less developed nation OR less developed nations OR less resourced country OR less resourced countries OR less resourced nation OR less resourced nations OR low resource country OR low resource countries OR low resource nation OR low resource nations OR low resource setting OR low resource settings OR low resourced country OR low resourced countries OR low resourced nation OR low resourced nations OR low resourced setting OR low resourced settings OR third world country OR third world countries OR third world nation OR third world nations OR Africa OR Asia OR Central America OR Latin America OR South America) OR MH "Afghanistan" OR MH "Albania" OR MH "Algeria" OR MH "Angola" OR MH "Argentina" OR MH "Armenia" OR MH "Azerbaijan" OR MH "Bangladesh" OR MH "Byelarus" OR MH "Belize" OR MH "Benin" OR MH "Bhutan" OR MH "Bolivia" OR MH "Bosnia-Herzegovina" OR MH "Botswana" OR MH "Brazil" OR MH "Burkina Faso" OR MH "Cape Verde" OR MH "Cambodia" OR MH "Cameroon" OR MH "Central African Republic" OR MH "Chad" OR MH "China+" OR MH "Congo" OR MH "Costa Rica" OR MH "Cote d'Ivoire" OR MH "Cuba" OR MH "Djibouti" OR MH "Dominica" OR MH "Dominican Republic" OR MH "Ecuador" OR MH "Egypt" OR MH "El Salvador" OR MH "Equatorial Guinea" OR MH "Eritrea" OR MH "Ethiopia" OR MH "Melanesia+" OR MH "Gabon" OR MH "Gambia" OR MH "Georgia (Republic)" OR MH "Ghana" OR MH "Guatemala" OR MH "Guinea" OR MH "Guinea-Bissau" OR MH "Guyana" OR MH "Haiti" OR MH "Honduras" OR MH "India" OR MH "Indonesia" OR MH "Iran" OR MH "Iraq" OR MH "Jamaica" OR MH "Jordan" OR MH "Kazakhstan" OR MH "Kenya" OR MH "Yugoslavia+" OR MH "Kyrgyzstan" OR MH "Laos" OR MH "Lebanon" OR MH "Lesotho" OR MH "Liberia" OR MH "Libya" OR MH "Madagascar" OR MH "Malawi" OR MH "Malaysia" OR MH "Indian Ocean Islands+" OR MH "Mali" OR MH "Micronesia+" OR MH "Mauritania" OR MH "Mexico" OR MH "Moldova" OR MH "Mongolia" OR MH "Morocco" OR MH "Mozambique" OR MH "Myanmar" OR MH "Namibia" OR MH "Nepal" OR MH "Nicaragua" OR MH "Niger" OR MH "Nigeria" OR MH "Macedonia (Republic)" OR MH "Pakistan" OR MH "Papua New Guinea" OR MH "Paraguay" OR MH "Peru" OR MH "Philippines" OR MH "Russia" OR MH "Rwanda" OR MH "Samoa+" OR MH "Senegal" OR MH "Serbia" OR MH "Sierra Leone" OR MH "Somalia" OR MH "South Africa" OR MH "Sudan" OR MH "Sri Lanka" OR MH "Suriname" OR MH "Syria" OR MH "Tajikistan" OR MH "Tanzania" OR MH "Thailand" OR MH "Timor" OR MH "Togo" OR MH "Polynesia+" OR MH "Tunisia" OR MH "Turkey" OR MH "Turkmenistan" OR MH "Uganda" OR MH "Ukraine" OR MH "Uzbekistan" OR MH "Vietnam" OR MH "Middle East+" OR MH "Yemen" OR MH "Zimbabwe" OR TI (Afghanistan OR Albania OR Albanian OR Algeria OR Algerian OR Angola OR Angolan OR Argentina OR Argentinian OR Armenia OR Armenian OR Azerbaijan OR Bangladesh OR Belarus OR Byelarus OR Belorussia OR Belorussian OR Byelorussia OR Byelorussian OR Belize OR British Honduras OR Benin OR Bhutan OR Bolivia OR Bolivian OR Bosnia OR Bosnian OR Herzegovina OR Hercegovina OR Bosnia-Herzegovina OR Botswana OR Bechuanaland OR Brazil OR Brazilian OR Bulgaria OR Bulgarian OR Burkina Faso OR Burundi OR Urundi OR Cabo Verde OR Cape Verde OR Cambodia OR Cambodian OR Cameroon OR Central African Republic OR Chad OR Chadian OR China OR Chinese OR Colombia OR Colombian OR Comoros OR Comoro Islands OR Congo OR Costa Rica OR Costa Rican OR Cote d'Ivoire OR Ivory Coast OR Cuba OR Cuban OR Djibouti OR Dominica OR Dominican OR Dominican Republic OR Ecuador OR Egypt OR Egyptian OR El Salvador OR Salvadoran OR Equatorial Guinea OR Eritrea OR Eritrean OR Eswatini OR Ethiopia OR Ethiopian OR Fiji OR Gabon OR Gabonese OR Gambia OR Gambian OR Georgia OR Georgian OR Ghana OR Grenada OR Guatemala OR Guatemalan OR Guinea OR Guinean OR Guinea-Bissau OR Guyana OR Haiti OR Haitian OR Honduras OR India OR Indian OR Indonesia OR Indonesian OR Iran OR Iraq OR Jamaica OR Jamaican OR Jordan OR Kazakhstan OR Kenya OR Kenyan OR Kiribati OR Kosovo OR Kyrgyzstan OR Laos OR Lao PDR OR "Lao People’s Democratic Republic" OR Lebanon OR Lebanese OR Lesotho OR Liberia OR Liberian OR Libya OR Libyan OR Madagascar OR Malawi OR Malaysia OR Malaysian OR Maldives OR Mali OR Micronesia OR Micronesian OR Marshall Islands OR Mauritania OR Mauritanian OR Mexico OR Moldova OR Mongolia OR Mongolian OR Montenegro OR Morocco OR Moroccan OR Mozambique OR Myanmar OR Namibia OR Namibian OR Nepal OR Nicaragua OR Nicaraguan OR Niger OR Nigeria OR Nigerian OR North Macedonia OR Pakistan OR Pakistani OR Papua New Guinea OR Paraguay OR Peru OR Philippines OR Philippine OR Philippino OR Russia OR Russian OR Rwanda OR Rwandan OR Samoa OR Samoan OR Samoan Islands OR "Sao Tome and Principe" OR Senegal OR Serbia OR Serbian OR Sierra Leone OR Melanesia OR Melanesian OR Solomon Islands OR Somalia OR Somali OR South Africa OR South African OR South Sudan OR Sri Lanka OR Sri Lankan OR Saint Lucia OR St Lucia OR St. Lucia OR Grenadines OR Sudan OR Sudanese OR Suriname OR Syria OR Syrian OR Tajikistan OR Tadjikistan OR Tadzhikistan OR Tadzhik OR Tanzania OR Tanzanian OR Thailand OR Timor-Leste OR Togo OR Tonga OR Tunisia OR Turkey OR Turkish OR Turkmenistan OR Tuvalu OR Uganda OR Ugandan OR Ukraine OR Ukrainian OR Uzbekistan OR Uzbek OR Vanuatu OR Vietnam OR Viet Nam OR Vietnamese OR Middle East OR Middle Eastern OR West Bank OR Gaza OR Yemen OR Zambia OR Zambian OR Zimbabwe) OR AB (Afghanistan OR Albania OR Albanian OR Algeria OR Algerian OR Angola OR Angolan OR Argentina OR Argentinian OR Armenia OR Armenian OR Azerbaijan OR Bangladesh OR Belarus OR Byelarus OR Belorussia OR Belorussian OR Byelorussia OR Byelorussian OR Belize OR British Honduras OR Benin OR Bhutan OR Bolivia OR Bolivian OR Bosnia OR Bosnian OR Herzegovina OR Hercegovina OR Bosnia-Herzegovina OR Botswana OR Bechuanaland OR Brazil OR Brazilian OR Bulgaria OR Bulgarian OR Burkina Faso OR Burundi OR Urundi OR Cabo Verde OR Cape Verde OR Cambodia OR Cambodian OR Cameroon OR Central African Republic OR Chad OR Chadian OR China OR Chinese OR Colombia OR Colombian OR Comoros OR Comoro Islands OR Congo OR Costa Rica OR Costa Rican OR Cote d'Ivoire OR Ivory Coast OR Cuba OR Cuban OR Djibouti OR Dominica OR Dominican OR Dominican Republic OR Ecuador OR Egypt OR Egyptian OR El Salvador OR Salvadoran OR Equatorial Guinea OR Eritrea OR Eritrean OR Eswatini OR Ethiopia OR Ethiopian OR Fiji OR Gabon OR Gabonese OR Gambia OR Gambian OR Georgia OR Georgian OR Ghana OR Grenada OR Guatemala OR Guatemalan OR Guinea OR Guinean OR Guinea-Bissau OR Guyana OR Haiti OR Haitian OR Honduras OR India OR Indian OR Indonesia OR Indonesian OR Iran OR Iraq OR Jamaica OR Jamaican OR Jordan OR Kazakhstan OR Kenya OR Kenyan OR Kiribati OR Kosovo OR Kyrgyzstan OR Laos OR Lao PDR OR "Lao People’s Democratic Republic" OR Lebanon OR Lebanese OR Lesotho OR Liberia OR Liberian OR Libya OR Libyan OR Madagascar OR Malawi OR Malaysia OR Malaysian OR Maldives OR Mali OR Micronesia OR Micronesian OR Marshall Islands OR Mauritania OR Mauritanian OR Mexico OR Moldova OR Mongolia OR Mongolian OR Montenegro OR Morocco OR Moroccan OR Mozambique OR Myanmar OR Namibia OR Namibian OR Nepal OR Nicaragua OR Nicaraguan OR Niger OR Nigeria OR Nigerian OR North Macedonia OR Pakistan OR Pakistani OR Papua New Guinea OR Paraguay OR Peru OR Philippines OR Philippine OR Philippino OR Russia OR Russian OR Rwanda OR Rwandan OR Samoa OR Samoan OR Samoan Islands OR "Sao Tome and Principe" OR Senegal OR Serbia OR Serbian OR Sierra Leone OR Melanesia OR Melanesian OR Solomon Islands OR Somalia OR Somali OR South Africa OR South African OR South Sudan OR Sri Lanka OR Sri Lankan OR Saint Lucia OR St Lucia OR St. Lucia OR Grenadines OR Sudan OR Sudanese OR Suriname OR Syria OR Syrian OR Tajikistan OR Tadjikistan OR Tadzhikistan OR Tadzhik OR Tanzania OR Tanzanian OR Thailand OR Timor-Leste OR Togo OR Tonga OR Tunisia OR Turkey OR Turkish OR Turkmenistan OR Tuvalu OR Uganda OR Ugandan OR Ukraine OR Ukrainian OR Uzbekistan OR Uzbek OR Vanuatu OR Vietnam OR Viet Nam OR Vietnamese OR Middle East OR Middle Eastern OR West Bank OR Gaza OR Yemen OR Zambia OR Zambian OR Zimbabwe)) AND (MH "Prevalence" OR MH “Incidence” OR MH “Epidemiology+” OR TI (prevalen* OR incidence OR incidences OR epidemiolog*) OR AB (prevalen* OR incidence OR incidences OR epidemiolog*)) AND (MH "Pediatrics+" OR MH “Child+” OR MH “Infant+” OR MH “Adolescence+” OR TI (pediatric OR pediatrics OR paediatric OR paediatrics OR children* OR child OR childs OR child’s OR childhood OR infant OR infants OR infantile OR infancy OR newborn OR newborns OR neonat* OR baby OR babies OR toddler OR toddlers OR preschool* OR pre-school* OR school age* OR schoolchildren OR teen* OR youth OR youths OR juvenile OR juveniles OR puberty OR pubescent OR adolescen* OR preadolescen* OR pre-adolescen* OR boy OR boys OR girl OR girls) OR AB (pediatric OR pediatrics OR paediatric OR paediatrics OR pediatrician OR pediatricians OR paediatrician OR paediatricians OR children* OR child OR childs OR child’s OR childhood OR infant OR infants OR infantile OR infancy OR newborn OR newborns OR neonat* OR baby OR babies OR toddler OR toddlers OR preschool* OR pre-school* OR school age* OR schoolchildren OR teen* OR youth OR youths OR juvenile OR juveniles OR puberty OR pubescent OR adolescen* OR preadolescen* OR pre-adolescen* OR boy OR boys OR girl OR girls)) AND MH Human AND DT 18830101-20201201 NOT PT (Review OR Case Study OR Proceedings)

**322 Results**

Excluded Reviews, Case Studies, and Conference/Meeting Abstracts

Limited to publication dates 1/7/2022 and prior

Limited to Humans

**PsycINFO (ProQuest):** <http://www.library.vanderbilt.edu/eres?id=143>

**Date Last Searched: 1/7/2022**

(MAINSUBJECT.EXACT.EXPLODE("Chronic Pain") OR ((MAINSUBJECT.EXACT.EXPLODE("Pain") OR TI,AB(pain OR pains OR painful* OR ache OR aches OR aching OR backache OR backaches OR headache OR headaches)) AND (NOFT(chronic) OR TI,AB(chronic* OR persist* OR recurrent OR recurring OR reoccurrent OR reoccurring)))) AND (MAINSUBJECT.EXACT.EXPLODE("Developing Countries") OR TI,AB(LMIC OR LMICs OR LIC OR LICs OR MIC OR MICs OR low income country OR low income countries OR low income nation OR low income nations OR low income setting OR low income settings OR low income economy OR low income economies OR lower income country OR lower income countries OR lower income nation OR lower income nations OR lower income economy OR lower income economies OR middle income country OR middle income countries OR middle income nation OR middle income nations OR middle income setting OR middle income settings OR middle income economy OR middle income economies OR developing country OR developing countries OR developing nation OR developing nations OR developing economy OR developing economies OR under developed country OR under developed countries OR under developed nation OR under developed nations OR less developed country OR less developed countries OR less developed nation OR less developed nations OR less resourced country OR less resourced countries OR less resourced nation OR less resourced nations OR low resource country OR low resource countries OR low resource nation OR low resource nations OR low resource setting OR low resource settings OR low resourced country OR low resourced countries OR low resourced nation OR low resourced nations OR low resourced setting OR low resourced settings OR third world country OR third world countries OR third world nation OR third world nations OR Africa OR Asia OR Central America OR Latin America OR South America OR Afghanistan OR Albania OR Albanian OR Algeria OR Algerian OR Angola OR Angolan OR Argentina OR Argentinian OR Armenia OR Armenian OR Azerbaijan OR Bangladesh OR Belarus OR Byelarus OR Belorussia OR Belorussian OR Byelorussia OR Byelorussian OR Belize OR British Honduras OR Benin OR Bhutan OR Bolivia OR Bolivian OR Bosnia OR Bosnian OR Herzegovina OR Hercegovina OR Bosnia-Herzegovina OR Botswana OR Bechuanaland OR Brazil OR Brazilian OR Bulgaria OR Bulgarian OR Burkina Faso OR Burundi OR Urundi OR Cabo Verde OR Cape Verde OR Cambodia OR Cambodian OR Cameroon OR Central African Republic OR Chad OR Chadian OR China OR Chinese OR Colombia OR Colombian OR Comoros OR Comoro Islands OR Congo OR Costa Rica OR Costa Rican OR Cote d'Ivoire OR Ivory Coast OR Cuba OR Cuban OR Djibouti OR Dominica OR Dominican OR Dominican Republic OR Ecuador OR Egypt OR Egyptian OR El Salvador OR Salvadoran OR Equatorial Guinea OR Eritrea OR Eritrean OR Eswatini OR Ethiopia OR Ethiopian OR Fiji OR Gabon OR Gabonese OR Gambia OR Gambian OR Georgia OR Georgian OR Ghana OR Grenada OR Guatemala OR Guatemalan OR Guinea OR Guinean OR Guinea-Bissau OR Guyana OR Haiti OR Haitian OR Honduras OR India OR Indian OR Indonesia OR Indonesian OR Iran OR Iraq OR Jamaica OR Jamaican OR Jordan OR Kazakhstan OR Kenya OR Kenyan OR Kiribati OR Kosovo OR Kyrgyzstan OR Laos OR Lao PDR OR "Lao People’s Democratic Republic" OR Lebanon OR Lebanese OR Lesotho OR Liberia OR Liberian OR Libya OR Libyan OR Madagascar OR Malawi OR Malaysia OR Malaysian OR Maldives OR Mali OR Micronesia OR Micronesian OR Marshall Islands OR Mauritania OR Mauritanian OR Mexico OR Moldova OR Mongolia OR Mongolian OR Montenegro OR Morocco OR Moroccan OR Mozambique OR Myanmar OR Namibia OR Namibian OR Nepal OR Nicaragua OR Nicaraguan OR Niger OR Nigeria OR Nigerian OR North Macedonia OR Pakistan OR Pakistani OR Papua New Guinea OR Paraguay OR Peru OR Philippines OR Philippine OR Philippino OR Russia OR Russian OR Rwanda OR Rwandan OR Samoa OR Samoan OR Samoan Islands OR "Sao Tome and Principe" OR Senegal OR Serbia OR Serbian OR Sierra Leone OR Melanesia OR Melanesian OR Solomon Islands OR Somalia OR Somali OR South Africa OR South African OR South Sudan OR Sri Lanka OR Sri Lankan OR Saint Lucia OR St Lucia OR St. Lucia OR Grenadines OR Sudan OR Sudanese OR Suriname OR Syria OR Syrian OR Tajikistan OR Tadjikistan OR Tadzhikistan OR Tadzhik OR Tanzania OR Tanzanian OR Thailand OR Timor-Leste OR Togo OR Tonga OR Tunisia OR Turkey OR Turkish OR Turkmenistan OR Tuvalu OR Uganda OR Ugandan OR Ukraine OR Ukrainian OR Uzbekistan OR Uzbek OR Vanuatu OR Vietnam OR Viet Nam OR Vietnamese OR Middle East OR Middle Eastern OR West Bank OR Gaza OR Yemen OR Zambia OR Zambian OR Zimbabwe)) AND (MAINSUBJECT.EXACT.EXPLODE("Epidemiology") OR TI,AB(prevalen* OR incidence OR incidences OR epidemiolog*)) AND (MAINSUBJECT.EXACT.EXPLODE("Pediatrics") OR MAINSUBJECT.EXACT.EXPLODE("Neonatal Period") OR MAINSUBJECT.EXACT.EXPLODE("Puberty") OR MAINSUBJECT.EXACT.EXPLODE("Early Adolescence") OR MAINSUBJECT.EXACT.EXPLODE("Childhood Development") OR MAINSUBJECT.EXACT.EXPLODE("Adolescent Development") OR TI,AB(pediatric OR pediatrics OR paediatric OR paediatrics OR children* OR child OR childs OR child’s OR childhood OR infant OR infants OR infantile OR infancy OR newborn OR newborns OR neonat* OR baby OR babies OR toddler OR toddlers OR preschool* OR pre-school* OR school age* OR schoolchildren OR teen* OR youth OR youths OR juvenile OR juveniles OR puberty OR pubescent OR adolescen* OR preadolescen* OR pre-adolescen* OR boy OR boys OR girl OR girls)) AND POP(Human) AND PD(18830101-20201201)

**45 Results**

Limited to publication dates 1/7/2022 and prior

Limited to Humans

No option to exclude Reviews, Case Studies, or Conference/Meeting Abstracts

**Web of Science:** <http://www.library.vanderbilt.edu/eres?id=1241>
**Date Last Searched: 1/7/2022**

(“chronic pain” OR ((pain OR pains OR painful* OR ache OR aches OR aching OR backache OR backaches OR headache OR headaches) AND (chronic* OR persist* OR recurrent OR recurring OR reoccurrent OR reoccurring))) AND (LMIC OR LMICs OR LIC OR LICs OR MIC OR MICs OR “low income country” OR “low income countries” OR “low income nation” OR “low income nations” OR “low income setting” OR “low income settings” OR “low income economy” OR “low income economies” OR “lower income country” OR “lower income countries” OR “lower income nation” OR “lower income nations” OR “lower income economy” OR “lower income economies” OR “middle income country” OR “middle income countries” OR “middle income nation” OR “middle income nations” OR “middle income setting” OR “middle income settings” OR “middle income economy” OR “middle income economies” OR “developing country” OR “developing countries” OR “developing nation” OR “developing nations” OR “developing economy” OR “developing economies” OR “under developed country” OR “under developed countries” OR “under developed nation” OR “under developed nations” OR “less developed country” OR “less developed countries” OR “less developed nation” OR “less developed nations” OR “less resourced country” OR “less resourced countries” OR “less resourced nation” OR “less resourced nations” OR “low resource country” OR “low resource countries” OR “low resource nation” OR “low resource nations” OR “low resource setting” OR “low resource settings” OR “low resourced country” OR “low resourced countries” OR “low resourced nation” OR “low resourced nations” OR “low resourced setting” OR “low resourced settings” OR “third world country” OR “third world countries” OR “third world nation” OR “third world nations” OR Africa OR Asia OR “Central America” OR “Latin America” OR “South America” OR Afghanistan OR Albania OR Albanian OR Algeria OR Algerian OR Angola OR Angolan OR Argentina OR Argentinian OR Armenia OR Armenian OR Azerbaijan OR Bangladesh OR Belarus OR Byelarus OR Belorussia OR Belorussian OR Byelorussia OR Byelorussian OR Belize OR “British Honduras” OR Benin OR Bhutan OR Bolivia OR Bolivian OR Bosnia OR Bosnian OR Herzegovina OR Hercegovina OR “Bosnia-Herzegovina” OR Botswana OR Bechuanaland OR Brazil OR Brazilian OR Bulgaria OR Bulgarian OR “Burkina Faso” OR Burundi OR Urundi OR “Cabo Verde” OR “Cape Verde” OR Cambodia OR Cambodian OR Cameroon OR “Central African Republic” OR Chad OR Chadian OR China OR Chinese OR Colombia OR Colombian OR Comoros OR “Comoro Islands” OR Congo OR “Costa Rica” OR “Costa Rican” OR “Cote d'Ivoire” OR “Ivory Coast” OR Cuba OR Cuban OR Djibouti OR Dominica OR Dominican OR “Dominican Republic” OR Ecuador OR Egypt OR Egyptian OR “El Salvador” OR Salvadoran OR “Equatorial Guinea” OR Eritrea OR Eritrean OR Eswatini OR Ethiopia OR Ethiopian OR Fiji OR Gabon OR Gabonese OR Gambia OR Gambian OR Georgia OR Georgian OR Ghana OR Grenada OR Guatemala OR Guatemalan OR Guinea OR Guinean OR “Guinea-Bissau” OR Guyana OR Haiti OR Haitian OR Honduras OR India OR Indian OR Indonesia OR Indonesian OR Iran OR Iraq OR Jamaica OR Jamaican OR Jordan OR Kazakhstan OR Kenya OR Kenyan OR Kiribati OR Kosovo OR Kyrgyzstan OR Laos OR “Lao PDR” OR "Lao People’s Democratic Republic" OR Lebanon OR Lebanese OR Lesotho OR Liberia OR Liberian OR Libya OR Libyan OR Madagascar OR Malawi OR Malaysia OR Malaysian OR Maldives OR Mali OR Micronesia OR Micronesian OR “Marshall Islands” OR Mauritania OR Mauritanian OR Mexico OR Moldova OR Mongolia OR Mongolian OR Montenegro OR Morocco OR Moroccan OR Mozambique OR Myanmar OR Namibia OR Namibian OR Nepal OR Nicaragua OR Nicaraguan OR Niger OR Nigeria OR Nigerian OR “North Macedonia” OR Pakistan OR Pakistani OR “Papua New Guinea” OR Paraguay OR Peru OR Philippines OR Philippine OR Philippino OR Russia OR Russian OR Rwanda OR Rwandan OR Samoa OR Samoan OR “Samoan Islands” OR "Sao Tome and Principe" OR Senegal OR Serbia OR Serbian OR “Sierra Leone” OR Melanesia OR Melanesian OR “Solomon Islands” OR Somalia OR Somali OR “South Africa” OR “South African” OR “South Sudan” OR “Sri Lanka” OR “Sri Lankan” OR “Saint Lucia” OR “St Lucia” OR “St. Lucia” OR Grenadines OR Sudan OR Sudanese OR Suriname OR Syria OR Syrian OR Tajikistan OR Tadjikistan OR Tadzhikistan OR Tadzhik OR Tanzania OR Tanzanian OR Thailand OR “Timor-Leste” OR Togo OR Tonga OR Tunisia OR Turkey OR Turkish OR Turkmenistan OR Tuvalu OR Uganda OR Ugandan OR Ukraine OR Ukrainian OR Uzbekistan OR Uzbek OR Vanuatu OR Vietnam OR “Viet Nam” OR Vietnamese OR “Middle East” OR “Middle Eastern” OR “West Bank” OR Gaza OR Yemen OR Zambia OR Zambian OR Zimbabwe) AND (prevalen* OR incidence OR incidences OR epidemiolog*) AND (pediatric OR pediatrics OR paediatric OR paediatrics OR children* OR child OR childs OR child’s OR childhood OR infant OR infants OR infantile OR infancy OR newborn OR newborns OR neonat* OR baby OR babies OR toddler OR toddlers OR preschool* OR “pre-school*” OR "school age*" OR schoolchildren OR teen* OR youth OR youths OR juvenile OR juveniles OR puberty OR pubescent OR adolescen* OR preadolescen* OR pre-adolescen* OR boy OR boys OR girl OR girls)

**484 Results**: Exclude Reviews and Conference Abstracts (Case Studies not listed as an option), Limit to publication dates 2022 and prior (cannot limit by month/day)

No option to limit to Humans

**Cochrane Library:** <https://ckm.vumc.org/ckm/diglib/ckmres.html?rid=6610>
**Date Last Searched: 1/29/2021**

(pain OR pains OR painful* OR ache OR aches OR aching OR backache* OR headache*) AND (chronic* OR persist* OR recurrent OR recurring OR reoccurrent OR reoccurring) AND (LMIC OR LMICs OR LIC OR LICs OR MIC OR MICs OR Africa OR Asia OR “Central America” OR “Latin America” OR “South America” OR “Middle East” OR ((“low income” OR “lower income” OR “middle income” OR developing OR “under developed” OR “less developed” OR “less resourced” OR “low resource” OR “low resourced” OR “third world”) AND (countr* OR nation* OR econom* OR setting*))) AND (prevalen* OR incidence* OR epidemiolog*) AND (pediatric* OR paediatric* OR child OR children* OR infan* OR newborn OR newborns OR neonat* OR baby OR babies OR toddler OR toddlers OR preschool* OR pre-school* OR teen* OR adolescen* OR preadolescen* OR pre-adolescen* OR boy* OR girl*)

**15 Cochrane Reviews, 98 Trials**: Limit to publication dates 2022 and prior

No option to limit to Humans

**WHO Global Index Medicus:** <https://www.globalindexmedicus.net/>

**Date Last Searched: 2/1/2021**

(pain OR pains OR painful* OR ache OR aches OR aching OR backache* OR headache*) AND (chronic* OR persist* OR recurrent OR recurring OR reoccurrent OR reoccurring) AND (LMIC OR LMICs OR LIC OR LICs OR MIC OR MICs OR Africa OR African OR Asia OR Asian OR “Central America” OR “Central American” OR “Latin America” OR “Latin American” OR “South America” OR “South American” OR “Middle East” OR “Middle Eastern" OR ((“low income” OR “lower income” OR “middle income” OR developing OR “under developed” OR “less developed” OR “less resourced” OR “low resource” OR “low resourced” OR “third world”) AND (countr* OR nation* OR econom* OR setting*))) AND (prevalen* OR incidence* OR epidemiolog*) AND (pediatric* OR paediatric* OR child OR children* OR infan* OR newborn OR newborns OR neonat* OR baby OR babies OR toddler OR toddlers OR preschool* OR pre-school* OR teen* OR adolescen* OR preadolescen* OR pre-adolescen* OR boy* OR girl*)

**303 Results** when limited to publication dates 2022 and prior

**Number of Citations:**

**Before De-Duplicating:**

PubMed: 921

Embase: 1764

CINAHL: 322

PsycINFO: 45

Web of Science: 484

Cochrane Library: 113

WHO Global Index Medicus: 303

Total: 3952

**After De-Duplicating:**

PubMed: 919

Embase: 1206

CINAHL: 157

PsycINFO: 16

Web of Science: 209

Cochrane Library: 98

WHO Global Index Medicus: 270

Total: 2875

**Number of Duplicates Removed:**

PubMed: 2

Embase: 558

CINAHL: 165

PsycINFO: 29

Web of Science: 275

Cochrane Library: 15

WHO Global Index Medicus: 33

Total: 1077
