# Supplementary material for: Paediatric chronic pain prevalence in low- and middle-income countries: A systematic review and meta-analysis
Source: eClinicalMedicine. 2022 Feb 12;45:101296. doi: 10.1016/j.eclinm.2022.101296 (PMC8850335; doi:10.1016/j.eclinm.2022.101296)
Supplement: Supplementary file 2 [file mmc2.docx]

Appendix 2. List of studies excluded at full-text screening stage.

| **Study** | **Pubmed ID or DOI** | **Reason for Exclusion** |
| --- | --- | --- |
| Young-Il, Rho (2017) | <https://doi.org/10.5124/jkma.2017.60.2.112> | Chronicity defined as 1 month. |
| Kitai et al (1997) | 9372464 | Not on chronic temporomandibular disorders. |
| Boey et al (2001) | 11207895 | Population already included. |
| Kong et al (2001) | 11406673 | Not on chronic pain. |
| Boey and Goh (2001) | 11595243 | Population already included. |
| Ozge et al (2003) | 12603371 | Does not define chronicity. |
| Shehab et al (2004) | 15073426 | Not on chronic pain. |
| Bugdayci et al (2005) | 15910458 | Not on chronic pain. |
| Chakravarty, A. (2005) | 16162256 | Chronic defined as >6 weeks |
| Chung and Wong (2007) | 17371410 | Not on chronic pain. |
| Knezević-Pogancev, M (2008) | 18716408 | Does not define chronicity of recurrent headache. |
| Al-Tulaihi and Al-Jumah (2009) | 19139785 | Does not define chronic pain. |
| Bar-Dayan et al (2010) | 20042693 | Not on chronic pain. |
| Arruda et al (2010) | 21093733 | Population already included. |
| IzmaÄ­lova et al (2011) | 22250391 | Full-text not available. |
| Benbir et al (2012) | 23364776 | Does not include chronic headache data on children and adolescents. |
| Lu et al (2013) | 23776112 | Study is on incidence rather than prevalence of chronic headache. |
| Queiroz et al (2013) | 23861946 | Not on chronic pain |
| Castro et al (2013 | 24160214 | Does not define chronicity of headache. |
| Zhao et al (2014) | 25623316 | Chronic pain defined as “lasting over 6 hours a single time or short time with high-frequency over 2-3 one day, and this bad situation has happened more than 3 times in one month.” |
| Chiwaridzo and Naidoo (2016) | 26148568 | Recurrent low back pain was defined as “2 times over the past year with each episode lasting at least 24 hours.” |
| Franco-Micheloni et al (2015) | 26244432 | Population already included. |
| Chiwaridzo and Naidoo (2015) | 26645723 | Recurrent low back pain was defined as “2 times over the past year with each episode lasting at least 24 hours.” |
| Hikita, T. (2016) | 27460403 | Conducted in high-income country. |
| Azabagic et al (2016) | 27482154 | Does not define chronicity. |
| Boey and Yap (1999) | 10404456 | Population already included. |
| Perquin et al (2000) | 10863045 | Conducted in high-income country. |
| Sahlu et al (2019) | 30677038 | Worsening severe chronic headache was defined as “symptoms occurring more than once a week for more than 2 weeks.” |
| de Melo Junior et al (2019) | 30735506 | Definition of chronicity does not include recurring or lasting for at least 3 months. |
| Schwertner et al (2020) | 31356188 | Does not define chronic pain. |
| Boey et al (2003) | 12887665 | Population already included. |
| Da Silva Jr et al (2010) | 20163479 | Not restricted to pediatric population |
| Koh et al (2014) | 365756445 | Does not define chronic pain. |
| Zhao et al (2014) | 25623316 | Not on chronic pain. |
| Mehta, S. (2015) | 25878735 | Not on chronic pain. |
| Alashqar et al (2020) | 32765391 | Does not define chronic pain. |
| Adebayo et al (2020) | 32021403 | Not on chronic pain. |
| Fifi et al (2020) | 32698030 | Not on chronic pain |
| Wang et al (2004) | 14687014 | Study based in Taiwan, China, which is considered a high-income economy by the World Bank. |
| Knezevic-Pogancev et al. (2011) | 21849942 | Not on chronic pain. |
| Arruda and Bigal (2012) | 23109652 | Population already included. |
| Zhang et al (2015) | 26296558 | Chronic pain defined as “lasting over 6 hours a single time or short time with high-frequency over 2-3 one day, and this bad situation has happened more than 3 times in one month.” |
