# Supplementary material for: Paediatric chronic pain prevalence in low- and middle-income countries: A systematic review and meta-analysis
Source: eClinicalMedicine. 2022 Feb 12;45:101296. doi: 10.1016/j.eclinm.2022.101296 (PMC8850335; doi:10.1016/j.eclinm.2022.101296)

Appendix 3: Risk of bias assessment: A) risk of bias in indivisual studies. B) summary risk of bias asseessment.

A.


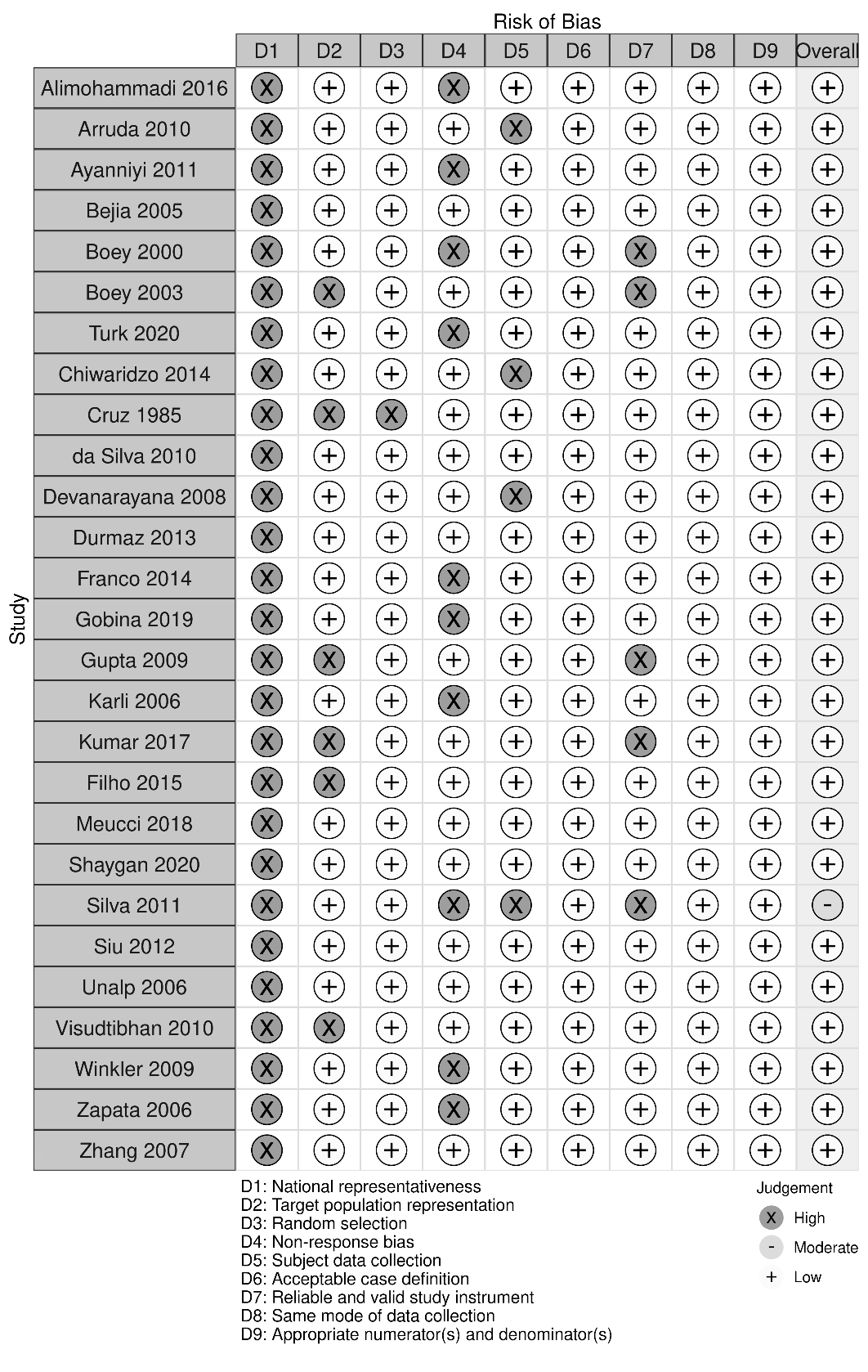


B.


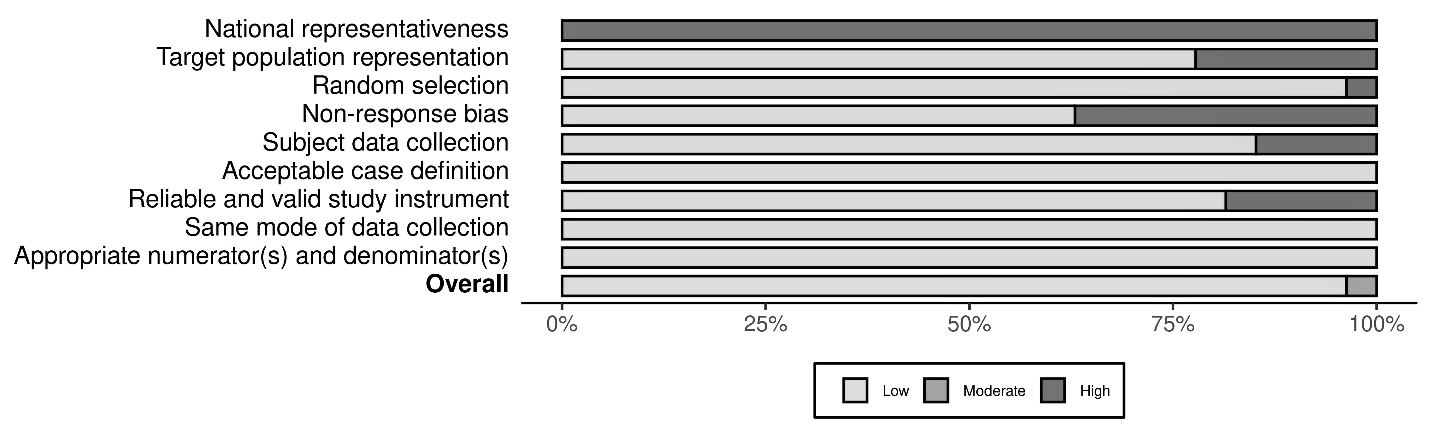

Supplement: Supplementary file 3 [file mmc3.docx]
