# Supplementary material for: Paediatric chronic pain prevalence in low- and middle-income countries: A systematic review and meta-analysis
Source: eClinicalMedicine. 2022 Feb 12;45:101296. doi: 10.1016/j.eclinm.2022.101296 (PMC8850335; doi:10.1016/j.eclinm.2022.101296)
Supplement: Supplementary file 4 [file mmc4.docx]

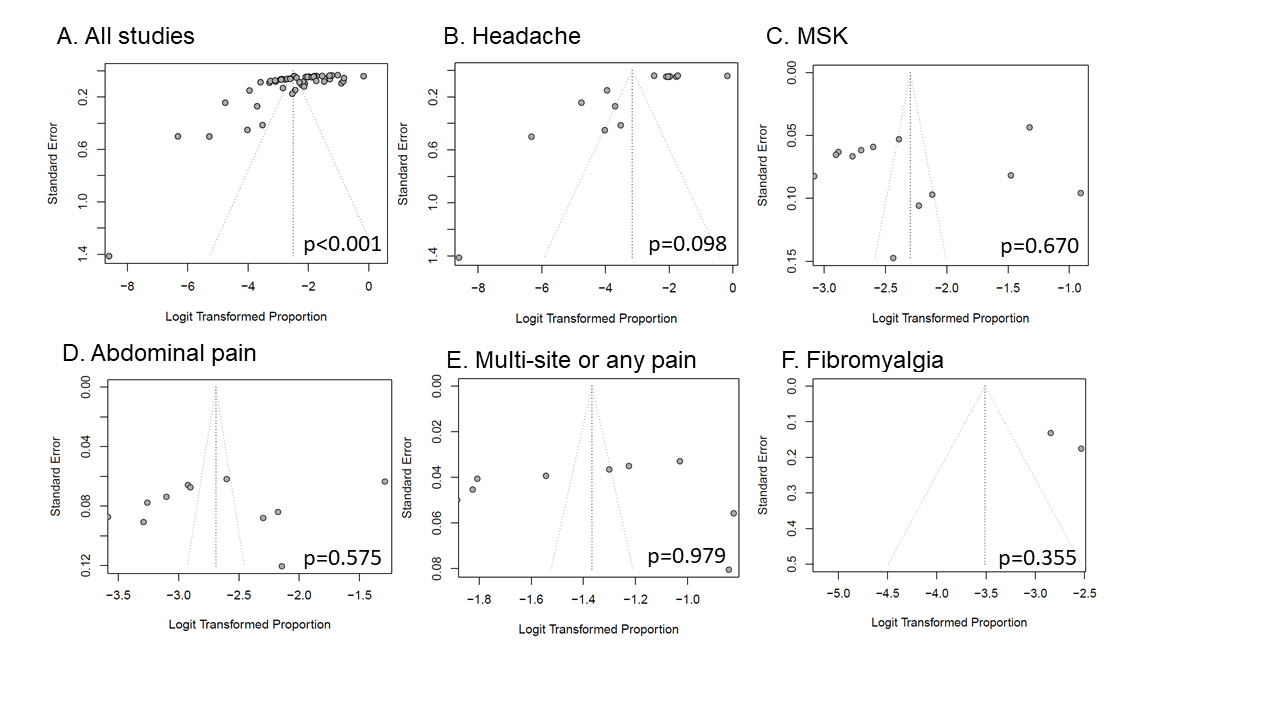


Appendix 4. Funnel plots of A) all included studies, B) headache only, C) musculoskeletal pain only, D) abdominal pain only, E) generalized or multi-site or any chronic pain, F) fibromyalgia only. A p-value <0.05 (Egger test) suggests significant publication bias.
